# Surface complexation model for strontium sorption to amorphous silica and goethite

**DOI:** 10.1186/1467-4866-9-2

**Published:** 2008-01-18

**Authors:** Susan A Carroll, Sarah K Roberts, Louise J Criscenti, Peggy A O'Day

**Affiliations:** 1 Chemistry, Materials, Earth, and Life Sciences Directorate, Lawrence Livermore National Laboratory, Livermore, California, USA; 2 Geochemistry Department, Sandia National Laboratories, Albuquerque, New Mexico, USA; 3 School of Natural Sciences, University of California, Merced, California, USA

## Abstract

Strontium sorption to amorphous silica and goethite was measured as a function of pH and dissolved strontium and carbonate concentrations at 25°C. Strontium sorption gradually increases from 0 to 100% from pH 6 to 10 for both phases and requires multiple outer-sphere surface complexes to fit the data. All data are modeled using the triple layer model and the site-occupancy standard state; unless stated otherwise all strontium complexes are mononuclear. Strontium sorption to amorphous silica in the presence and absence of dissolved carbonate can be fit with tetradentate Sr^2+ ^and SrOH^+ ^complexes on the *β*-plane and a monodentate Sr^2+^complex on the diffuse plane to account for strontium sorption at low ionic strength. Strontium sorption to goethite in the absence of dissolved carbonate can be fit with monodentate and tetradentate SrOH^+ ^complexes and a tetradentate binuclear Sr^2+ ^species on the *β*-plane. The binuclear complex is needed to account for enhanced sorption at hgh strontium surface loadings. In the presence of dissolved carbonate additional monodentate Sr^2+ ^and SrOH^+ ^carbonate surface complexes on the *β*-plane are needed to fit strontium sorption to goethite. Modeling strontium sorption as outer-sphere complexes is consistent with quantitative analysis of extended X-ray absorption fine structure (EXAFS) on selected sorption samples that show a single first shell of oxygen atoms around strontium indicating hydrated surface complexes at the amorphous silica and goethite surfaces.

Strontium surface complexation equilibrium constants determined in this study combined with other alkaline earth surface complexation constants are used to recalibrate a predictive model based on Born solvation and crystal-chemistry theory. The model is accurate to about 0.7 log K units. More studies are needed to determine the dependence of alkaline earth sorption on ionic strength and dissolved carbonate and sulfate concentrations for the development of a robust surface complexation database to estimate alkaline earth sorption in the environment.

## Background

Ion sorption to mineral and amorphous solids has long been recognized as a process that controls the composition of trace elements in water. This process is particularly important for the transport of contaminants in the Earth's surface environment where sorption may retard transport by removing the contaminant from a mobile aqueous phase to a more stationary solid phase. Efforts to describe sorption in complex geological settings has evolved from a purely empirical approach in which distribution coefficients (K_d_) are a measure of the total amount of specific ion between the solid and aqueous phases for a complex solution and solid matrix specific to a contaminated site. Although this approach provides a direct measure of the ability of the solid matrix to sequester the contaminant from a specific solution, its empirical nature does not allow it to be applied outside of the specific parameters of the contaminated site. Another approach measures thermodynamic surface complexation constants which describe sorption as a series of specific reactions between dissolved ions and surface sites. In principle, thermodynamic data from several single mineral and element experiments can be combined to build a model that represents the complex systems found in nature, especially when coupled with aqueous speciation, mineral solubility, and kinetic databases. However an internally consistent surface complexation database for a wide range of ions and solids found in natural waters that capture the surface charge is still lacking [1]. Databases tend to adopt surface complexation models that account for surface charge if only one solid is available for uptake [2] and non-electrostatic models that ignore surface charge if multiple solids are available for uptake [3-5].

It is also important to be able to estimate adsorption constants for reactions between aqueous components and substrates for which data are lacking both so that surface complexation models can be applied to complex geochemical systems and to build a robust database. Towards this effort, Sverjensky and colleagues have applied the Born solvation and crystal-chemistry theory together with a site-occupancy standard state to develop a predictive triple layer surface complexation model for surface protonation, alkali, alkaline earth, heavy metal and anion sorption for aluminum, iron, manganese, silica, and titanium oxides/hydroxides [6-16].

We illustrate the need for a predictive surface complexation model by considering the role that strontium sorption may play for the safe disposal of radioactive waste. ^90^Sr is one of several fission products that are concentrated in nuclear weapon and energy reprocessing waste and may interact with several different oxides depending on the waste form and disposal environment. At the Hanford (Washington, USA) site, caustic liquid waste with high ^90^Sr concentrations was disposed in tanks buried below the subsurface. Some of these tanks have leaked into the subsurface, where the migration of strontium is dependent on both its interaction with natural minerals and the reaction products formed from the interaction of the waste liquid with the subsurface fluvial-glacial sedimentary deposits [17-20]. Future disposal of ^90^Sr may include solid waste forms of cement, glass, or ceramics [21-24]. The long-term disposal of ^90^Sr depends not only on the stability of the waste form, but also on the sorption of strontium leached from the waste form to possible secondary phases, such as calcite, amorphous silica, iron hydroxides and rutile produced by the degradation of the waste form and corrosion of steel canisters containing the waste.

In this paper new strontium sorption data to amorphous silica and goethite collected over a range of total strontium concentrations, pH, and dissolved carbonate concentrations are described using a surface complexation model that builds on and further calibrates Sverjensky's [16] predictive model for alkaline earth sorption. Surface complexation reactions are constrained with structural information inferred from spectroscopic analysis of strontium at the mineral-solution interface (this study, [25-28]).

## Experimental methods

### Starting materials

For experiments conducted in the absence of CO_2_, all reagents were prepared with freshly distilled and deionized water collected under a nitrogen atmosphere using a portable microwave still. The water was then transferred to a nitrogen atmosphere glove box and used to make FeCl_3_, SrCl_2_, KOH, and NaOH stock solutions from solids that were purged for 20 to 30 minutes under a nitrogen stream and weighed in the nitrogen atmosphere glove box.

For experiments conducted in the presence of CO_2_, stock solutions were made by dissolving reagent grade NaCl, SrCl_2_, FeCl_3 _and Fe(NO_3_)_3 _solids in distilled and deionized water. Commercial high purity NaOH, HNO_3_, and HCl stock solutions were also used to adjust the pH of the sorption experiments. Solutions were stored in sealed containers and were not continually exposed to the atmosphere. We define dissolved carbonate as the sum of dissolved aqueous carbon species in this paper. Sources for dissolved carbonate in the sorption experiments include diffusion of atmospheric CO_2 _when the suspensions were prepared, dissolved carbonate present in the NaOH stock solution used to adjust solution pH, and possibly carbonate sorbed to goethite when synthesized at atmospheric *p*CO_2_.

The amorphous silica used in the sorption experiments was synthetic silica gel (Mallinckrodt Silica), 100–200 mesh, (lot # 6512), with an average pore diameter of 150 Å. The gel was repeatedly cleaned ultrasonically with distilled and deionized water until the suspension yielded a clear supernatant after 10 minutes of settling. Cleaned gel was dried at 40°C for 24 hours and stored in a plastic container at room temperature. Surface area was 277 m^2 ^g^-1 ^determined by BET nitrogen gas adsorption. No effort was made to exclude atmospheric CO_2 _in the cleaning and drying procedure for amorphous silica.

Four lots of goethite were synthesized following protocols outlined in Schwertmann and Cornell [29]. For CO_2_-free sorption experiments, goethite (Lot 1) was synthesized using KOH and FeCl_3_•6H_2_0 in a nitrogen atmosphere from reagents dissolved in CO_2_-free distilled and deionized water. After initial formation of iron hydroxide, the suspension was purged with nitrogen for 60 hours at 70°C to transform the hydroxide to goethite. It was then rinsed repeatedly to remove chloride using dialysis tubing and CO_2_-free distilled and deionized water. Goethite was dried under a nitrogen stream at 40°C and stored in a nitrogen atmosphere glove box. Mineralogy was confirmed by XRD. For sorption experiments prepared in atmospheric CO_2_, goethite (Lots 2 and 3) was synthesized following the same protocol except that no effort was made to exclude CO_2_. For Lot 4, goethite was prepared from Fe(NO_3_)_3 _instead of FeCl_3_•6H_2_O and no effort was made to exclude CO_2_. Surface areas determined by BET nitrogen gas adsorption were: Lot 1 = 37.9 m^2 ^g^-1^; Lot 2 = 38.2 m^2 ^g^-1^; Lot 3 = 37.9 m^2 ^g^-1^; and Lot 4 = 27.7 m^2 ^g^-1^. An average surface area of 37.8 m^2 ^g^-1 ^was used to model the strontium sorption to goethite in NaCl solutions.

### Sorption experiments

Strontium sorption was measured in amorphous silica and goethite suspensions prepared in the presence and absence of atmospheric CO_2 _at 25°C from pH 6 to 10. Tables 1, 2, 3, 4, 5, 6 list total surface area, sorption atmosphere, ionic strength, and initial and final solution composition for each experiment. Amorphous silica or goethite was mixed with a freshly prepared SrCl_2_/NaCl or Sr(NO_3_)_2_/NaNO_3 _solution of the desired concentration in polycarbonate test tubes. After the pH was adjusted, the tubes were sealed, shaken vigorously by hand, and then reacted for 2 or 14 days in a constant temperature orbital-shaker water bath at 200 rpm. The 14-day experiments were conducted in goethite suspensions to see if additional reaction was needed to precipitate strontium carbonate at higher pH. At the end of the experiment, the final pH of each solution was measured, a sample (2.5 ml) was taken, filtered (4.1 nm pore size), acidified with high purity HCl or HNO_3 _to prevent SrCO_3 _precipitation, and analyzed for strontium by inductively coupled plasma atomic emission spectrometry (ICP-AES) (detection limit = 10^-7 ^molal; precision ± 2%). For many of the experiments prepared in the presence of atmospheric CO_2_, total dissolved carbonate was measured from a filtered sample using a carbon analyzer with an IR detector (detection limit = 5 × 10^-5 ^molal). With this technique, dissolved carbon is purged with 11 N phosphoric acid and nitrogen gas. The resulting concentrations are under saturated with respect to atmospheric CO_2 _at higher pH. Additional control experiments with no solid present were done to check for strontium sorption to vessel walls and for precipitation of SrCO_3_(s). Strontium sorption uncertainty is calculated from the analytical uncertainty of the initial and final solution concentrations and the uncertainty associated with a small amount of strontium inherent in the substrate measured from substrate control experiments. For the experiments prepared in the absence of CO_2_, preparation, sampling, and reagent storage were done in a nitrogen atmosphere glove box.

**Table 1:** Solution analyses for strontium sorption to amorphous silica in nitrogen atmosphere, I = 0.1 M NaCl and T = 25°C.

**Final pH**	**Surface Area m^**2 **^L^**-1**^**	**Initial [Sr] M**	**Final [Sr] M**	**%Sr orbed**	**Γ Sr sorbed Mol m^**-2**^**
Total Sr = 10^-3 ^M aged for 2 days				± 5%	

3.14	10934	9.99 × 10^-4^	9.71 × 10^-4^	2.8	2.57 × 10^-9^
3.89	10983	1.00 × 10^-3^	9.90 × 10^-4^	1.3	1.17 × 10^-9^
3.89	10983	1.00 × 10^-3^	9.88 × 10^-4^	1.5	1.38 × 10^-9^
5.42	11037	1.01 × 10^-3^	9.86 × 10^-4^	2.0	1.86 × 10^-9^
6.02	11075	1.01 × 10^-3^	9.83 × 10^-4^	2.5	2.32 × 10^-9^
6.33	11115	1.01 × 10^-3^	9.78 × 10^-4^	3.4	3.06 × 10^-9^
6.55	11095	1.01 × 10^-3^	9.97 × 10^-4^	1.5	1.40 × 10^-9^
7.12	11101	1.01 × 10^-3^	9.80 × 10^-4^	3.2	2.92 × 10^-9^
7.04	11035	1.01 × 10^-3^	9.74 × 10^-4^	3.4	3.15 × 10^-9^
7.42	10855	9.95 × 10^-4^	9.33 × 10^-4^	6.3	5.74 × 10^-9^
7.95	10705	9.80 × 10^-4^	8.40 × 10^-4^	14.2	1.30 × 10^-8^
8.62	10415	9.54 × 10^-4^	6.23 × 10^-4^	34.7	3.18 × 10^-8^
9.12	10088	9.21 × 10^-4^	4.16 × 10^-4^	54.8	5.00 × 10^-8^
9.52	9720	8.88 × 10^-4^	2.64 × 10^-4^	70.3	6.43 × 10^-8^

Total Sr = 10^-4 ^M aged for 2 days				± 5%	

4.20	11019	1.00 × 10^-4^	9.85 × 10^-5^	2.0	1.80 × 10^-10^
4.89	11005	1.01 × 10^-4^	9.88 × 10^-5^	1.7	1.60 × 10^-10^
5.50	11034	1.01 × 10^-4^	9.92 × 10^-5^	1.6	1.42 × 10^-10^
6.22	11013	1.01 × 10^-4^	1.01 × 10^-4^	0.4	3.93 × 10^-11^
6.33	11057	1.01 × 10^-4^	1.01 × 10^-4^	0.5	4.92 × 10^-11^
6.71	11110	1.01 × 10^-4^	9.97 × 10^-5^	1.7	1.56 × 10^-10^
6.62	11087	1.01 × 10^-4^	1.01 × 10^-4^	0.7	6.64 × 10^-11^
7.01	11086	1.01 × 10^-4^	9.84 × 10^-5^	2.5	2.30 × 10^-10^
7.46	10912	9.96 × 10^-5^	9.38 × 10^-5^	5.8	5.31 × 10^-10^
7.82	10809	9.86 × 10^-5^	8.75 × 10^-5^	11.3	1.03 × 10^-9^
8.54	10475	9.56 × 10^-5^	6.42 × 10^-5^	32.8	2.99 × 10^-9^
8.95	10257	9.39 × 10^-5^	4.89 × 10^-5^	47.9	4.38 × 10^-9^
9.43	9888	9.05 × 10^-5^	2.84 × 10^-5^	68.7	6.29 × 10^-9^

Total Sr = 10^-5 ^M aged for 2 days				± 5%	

6.82	11081	1.06 × 10^-5^	1.06 × 10^-5^	-0.5	-4.60 × 10^-12^
6.57	11086	1.06 × 10^-5^	1.04 × 10^-5^	1.5	1.41 × 10^-11^
6.57	11044	1.06 × 10^-5^	9.30 × 10^-6^	12.1	1.15 × 10^-10^
6.84	10939	1.05 × 10^-5^	1.03 × 10^-5^	1.5	1.42 × 10^-11^
7.22	10842	1.04 × 10^-5^	9.47 × 10^-6^	8.6	8.19 × 10^-11^
7.88	10672	1.02 × 10^-5^	8.33 × 10^-6^	18.3	1.74 × 10^-10^
7.91	10481	1.00 × 10^-5^	7.53 × 10^-6^	24.7	2.35 × 10^-10^
9.16	9584	9.14 × 10^-6^	2.40 × 10^-6^	73.8	7.03 × 10^-10^
9.74	8484	8.13 × 10^-6^	8.33 × 10^-7^	89.8	8.60 × 10^-10^

Total Sr = 1.5 10^-6 ^M aged for 2 days				± 10%	

6.32	11056	1.51 × 10^-6^	1.60 × 10^-6^	-5.5	-7.55 × 10^-12^
6.53	11013	1.51 × 10^-6^	1.56 × 10^-6^	-3.4	-4.65 × 10^-12^
6.82	11000	1.51 × 10^-6^	1.59 × 10^-6^	-5.0	-6.85 × 10^-12^
6.83	11032	1.51 × 10^-6^	1.55 × 10^-6^	-2.8	-3.86 × 10^-12^
7.06	10988	1.50 × 10^-6^	1.39 × 10^-6^	7.3	9.99 × 10^-12^
7.29	10900	1.49 × 10^-6^	1.45 × 10^-6^	2.7	3.74 × 10^-12^
8.65	9964	1.37 × 10^-6^	6.96 × 10^-7^	49.1	6.74 × 10^-11^
9.10	9534	1.31 × 10^-6^	3.77 × 10^-7^	71.2	9.76 × 10^-11^
9.16	9562	1.31 × 10^-6^	3.65 × 10^-7^	72.1	9.87 × 10^-11^
6.62	11041	1.52 × 10^-6^	1.51 × 10^-6^	0.6	7.93 × 10^-13^
6.59	11092	1.52 × 10^-6^	1.51 × 10^-6^	0.6	7.89 × 10^-13^
6.52	11036	1.52 × 10^-6^	1.48 × 10^-6^	2.7	3.71 × 10^-12^
6.77	10957	1.51 × 10^-6^	1.59 × 10^-6^	-5.6	-7.65 × 10^-12^
7.30	10831	1.48 × 10^-6^	1.37 × 10^-6^	7.4	1.01 × 10^-11^
9.62	9014	1.24 × 10^-6^	1.26 × 10^-7^	89.8	1.23 × 10^-10^

Total Sr = 6 × 10 ^-7 ^M aged for 2 days				± 20%	

6.34	11073	6.10 × 10^-7^	6.91 × 10^-7^	-13.3	-7.33 × 10^-12^
6.65	11058	6.09 × 10^-7^	6.70 × 10^-7^	-10.0	-5.53 × 10^-12^
6.78	11046	6.08 × 10^-7^	6.41 × 10^-7^	-5.4	-2.99 × 10^-12^
6.83	11028	6.07 × 10^-7^	6.20 × 10^-7^	-2.1	-1.13 × 10^-12^
7.29	10862	5.98 × 10^-7^	6.01 × 10^-7^	-0.6	-3.08 × 10^-13^
7.2	10841	5.97 × 10^-7^	6.20 × 10^-7^	-3.8	-2.07 × 10^-12^
8.66	9955	5.48 × 10^-7^	2.50 × 10^-7^	54.4	3.00 × 10^-11^
9.06	9417	5.19 × 10^-7^	1.21 × 10^-7^	76.7	4.22 × 10^-11^
9.62	8977	4.94 × 10^-7^	4.34 × 10^-8^	91.2	5.02 × 10^-11^

**Table 2:** Solution analyses for strontium sorption to amorphous silica in air, I = 0.1 M NaCl and T = 25°C.

**Final pH**	**Surface Area m^**2 **^L^**-1**^**	**Initial [Sr] M**	**Final [Sr] M**	**%Sr sorbed**	**Γ Sr sorbed Mol m^**-2**^**	**Total Dissolved Carbonate M**
Total Sr = 10^-3 ^M aged for 2 days				± 5%		

6.67	11073	9.98 × 10^-4^	1.01 × 10^-3^	-1.5	-1.31 × 10^-9^	not measured
6.72	11098	1.00 × 10^-3^	1.02 × 10^-3^	-1.6	-1.43 × 10^-9^	not measured
6.80	11086	9.98 × 10^-4^	1.03 × 10^-3^	-3.6	-3.27 × 10^-9^	not measured
6.90	11060	9.94 × 10^-4^	1.02 × 10^-3^	-2.5	-2.21 × 10^-9^	not measured
7.19	11012	9.91 × 10^-4^	1.05 × 10^-3^	-6.1	-5.46 × 10^-9^	not measured
7.89	10724	9.66 × 10^-4^	9.35 × 10^-4^	3.2	2.89 × 10^-9^	not measured
8.32	10522	9.45 × 10^-4^	8.00 × 10^-4^	15.4	1.38 × 10^-8^	not measured
8.65	10237	9.23 × 10^-4^	5.28 × 10^-4^	42.7	3.85 × 10^-8^	not measured
9.58	9224	8.33 × 10^-4^	3.22 × 10^-5^	96.1	8.68 × 10^-8^	not measured
6.54	11016	1.00 × 10^-3^	1.02 × 10^-3^	-1.5	-1.36 × 10^-9^	1.72 × 10^-5^
6.61	11026	1.00 × 10^-3^	1.01 × 10^-3^	-0.4	-3.41 × 10^-10^	3.61 × 10^-5^
6.70	11004	9.97 × 10^-4^	1.01 × 10^-3^	-0.9	-8.38 × 10^-10^	3.34 × 10^-5^
6.73	11003	9.99 × 10^-4^	9.99 × 10^-4^	0.0	1.79 × 10^-12^	3.69 × 10^-6^
7.14	10961	9.93 × 10^-4^	9.69 × 10^-4^	2.4	2.19 × 10^-9^	2.67 × 10^-5^
7.72	10758	9.77 × 10^-4^	8.82 × 10^-4^	9.7	8.76 × 10^-9^	1.72 × 10^-4^
8.65	10327	9.36 × 10^-4^	5.85 × 10^-4^	37.5	3.40 × 10^-8^	3.52 × 10^-4^
8.98	10019	9.04 × 10^-4^	4.33 × 10^-4^	52.1	4.70 × 10^-8^	4.44 × 10^-4^
9.57	9565	8.65 × 10^-4^	2.20 × 10^-4^	74.6	6.75 × 10^-8^	5.64 × 10^-4^
7.98	10670	9.68 × 10^-4^	8.49 × 10^-4^	12.2	1.11 × 10^-8^	1.04 × 10^-4^
8.06	10590	9.68 × 10^-4^	8.60 × 10^-4^	11.1	1.01 × 10^-8^	8.95 × 10^-5^
8.82	10187	9.27 × 10^-4^	5.25 × 10^-4^	43.3	3.95 × 10^-8^	1.05 × 10^-4^
8.84	10272	9.27 × 10^-4^	5.05 × 10^-4^	45.5	4.11 × 10^-8^	1.54 × 10^-4^
8.85	10228	9.28 × 10^-4^	5.30 × 10^-4^	42.8	3.89 × 10^-8^	1.46 × 10^-4^
8.91	10160	9.27 × 10^-4^	5.14 × 10^-4^	44.6	4.07 × 10^-8^	2.13 × 10^-4^
9.16	9992	9.05 × 10^-4^	3.86 × 10^-4^	57.3	5.20 × 10^-8^	1.53 × 10^-4^
9.19	9967	9.06 × 10^-4^	3.91 × 10^-4^	56.8	5.17 × 10^-8^	1.52 × 10^-4^
9.26	9767	8.88 × 10^-4^	3.77 × 10^-4^	57.5	5.23 × 10^-8^	3.47 × 10^-5^
9.38	9961	9.00 × 10^-4^	3.40 × 10^-4^	62.2	5.62 × 10^-8^	2.40 × 10^-5^
9.44	9849	8.96 × 10^-4^	3.24 × 10^-4^	63.8	5.81 × 10^-8^	9.92 × 10^-5^
9.53	9822	8.88 × 10^-4^	2.83 × 10^-4^	68.2	6.16 × 10^-8^	7.95 × 10^-5^
9.54	9787	8.88 × 10^-4^	2.72 × 10^-4^	69.4	6.30 × 10^-8^	1.85 × 10^-4^
9.72	9461	8.58 × 10^-4^	2.29 × 10^-4^	73.3	6.65 × 10^-8^	1.82 × 10^-4^

Total Sr = 10^-4 ^M aged for 2 days				± 5%		

7.08	11079	1.00 × 10^-4^	1.04 × 10^-4^	-3.5	-3.15 × 10^-10^	not measured
6.41	11063	1.01 × 10^-4^	1.06 × 10^-4^	-5.1	-4.57 × 10^-10^	not measured
6.92	11002	1.00 × 10^-4^	1.09 × 10^-4^	-8.9	-8.11 × 10^-10^	not measured
7.02	10966	9.99 × 10^-5^	1.05 × 10^-4^	-5.3	-4.79 × 10^-10^	not measured
7.26	10922	9.88 × 10^-5^	9.88 × 10^-5^	0.0	4.05 × 10^-13^	not measured
7.50	10712	9.70 × 10^-5^	9.10 × 10^-5^	6.2	5.61 × 10^-10^	not measured
8.57	10078	9.14 × 10^-5^	5.30 × 10^-5^	42.2	3.81 × 10^-9^	not measured
9.33	9404	8.51 × 10^-5^	2.15 × 10^-5^	75.2	6.77 × 10^-9^	not measured
9.86	8585	7.81 × 10^-5^	9.13 × 10^-6^	88.7	8.03 × 10^-9^	not measured

Total Sr = 10^-5 ^M aged for 2 days				± 5%		

6.21	10913	1.05 × 10^-5^	1.07 × 10^-5^	-1.4	-1.39 × 10^-11^	not measured
6.40	11302	1.06 × 10^-5^	1.04 × 10^-5^	1.2	1.13 × 10^-11^	not measured
6.70	11458	1.06 × 10^-5^	1.03 × 10^-5^	2.0	1.88 × 10^-11^	not measured
7.15	11367	1.05 × 10^-5^	1.01 × 10^-5^	3.8	3.54 × 10^-11^	not measured
6.95	10918	1.05 × 10^-5^	1.04 × 10^-5^	1.0	9.75 × 10^-12^	not measured
7.60	10844	1.03 × 10^-5^	9.45 × 10^-6^	8.5	8.10 × 10^-11^	not measured
7.91	10811	1.03 × 10^-5^	8.71 × 10^-6^	15.1	1.43 × 10^-10^	not measured
8.92	10175	9.72 × 10^-6^	4.36 × 10^-6^	55.2	5.27 × 10^-10^	not measured
9.58	9544	9.12 × 10^-6^	1.85 × 10^-6^	79.7	7.62 × 10^-10^	not measured

Total Sr = 10^-6 ^M aged for 2 days				± 10%		

6.52	11049	1.59 × 10^-6^	1.65 × 10^-6^	-3.9	-5.60 × 10^-12^	not measured
6.64	11038	1.59 × 10^-6^	1.53 × 10^-6^	3.4	4.84 × 10^-12^	not measured
6.71	11025	1.58 × 10^-6^	1.50 × 10^-6^	5.4	7.78 × 10^-12^	not measured
6.81	11019	1.58 × 10^-6^	1.48 × 10^-6^	6.7	9.56 × 10^-12^	not measured
6.88	11030	1.58 × 10^-6^	1.51 × 10^-6^	4.5	6.42 × 10^-12^	not measured
7.62	10867	1.56 × 10^-6^	1.42 × 10^-6^	8.8	1.26 × 10^-11^	not measured
8.39	10590	1.52 × 10^-6^	1.13 × 10^-6^	25.3	3.64 × 10^-11^	not measured
9.06	10235	1.47 × 10^-6^	7.21 × 10^-7^	50.9	7.32 × 10^-11^	not measured
9.59	9873	1.42 × 10^-6^	3.89 × 10^-7^	72.5	1.04 × 10^-10^	not measured

Total Sr = 7 × 10^-7 ^M aged for 2 days				± 20%		

6.89	11022	6.81 × 10^-7^	6.46 × 10^-7^	5.1	3.14 × 10^-12^	not measured
6.50	11070	6.83 × 10^-7^	6.57 × 10^-7^	3.8	2.31 × 10^-12^	not measured
6.70	11016	6.80 × 10^-7^	6.82 × 10^-7^	-0.3	-2.12 × 10^-13^	not measured
6.43	11013	6.80 × 10^-7^	7.18 × 10^-7^	-5.6	-3.44 × 10^-12^	not measured
7.56	10685	6.59 × 10^-7^	5.63 × 10^-7^	14.7	9.05 × 10^-12^	not measured
7.73	10508	6.49 × 10^-7^	5.15 × 10^-7^	20.6	1.27 × 10^-11^	not measured
8.52	9978	6.16 × 10^-7^	3.34 × 10^-7^	45.7	2.82 × 10^-11^	not measured
9.05	9538	5.89 × 10^-7^	1.71 × 10^-7^	70.9	4.38 × 10^-11^	not measured
9.53	8920	5.50 × 10^-7^	8.10 × 10^-8^	85.3	5.26 × 10^-11^	not measured

**Table 3:** Solution analyses for strontium sorption to amorphous silica in air, I = 0.005 M NaCl and T = 25°C.

**Final pH**	**Surface Area m^**2 **^L^**-1**^**	**Initial [Sr] M**	**Final [Sr] M**	**%Sr sorbed**	**Γ Sr sorbed Mol m^**-2**^**	**Total Dissolved Carbonate M**
Total Sr = 10^-4 ^M aged for 2 days				± 5%		

6.85	10857	1.14 × 10^-4^	5.28 × 10^-5^	53.9	5.62 × 10^-9^	3.84 × 10^-5^
6.88	10983	1.16 × 10^-4^	5.04 × 10^-5^	56.7	5.94 × 10^-9^	3.93 × 10^-5^
7.16	10815	1.12 × 10^-4^	1.57 × 10^-5^	86.4	8.87 × 10^-9^	5.47 × 10^-5^
7.33	9931	1.05 × 10^-4^	2.87 × 10^-5^	72.9	7.64 × 10^-9^	5.80 × 10^-5^
7.74	9472	9.72 × 10^-5^	1.94 × 10^-5^	80.4	8.21 × 10^-9^	6.23 × 10^-5^
7.86	8468	8.92 × 10^-5^	1.81 × 10^-5^	80.0	8.39 × 10^-9^	2.45 × 10^-4^
7.97	8962	8.89 × 10^-5^	1.35 × 10^-5^	85.3	8.41 × 10^-9^	2.60 × 10^-4^
8.21	8502	8.41 × 10^-5^	7.74 × 10^-6^	91.3	8.98 × 10^-9^	2.21 × 10^-4^
8.36	8501	7.97 × 10^-5^	5.24 × 10^-6^	94.0	8.77 × 10^-9^	2.25 × 10^-4^
8.48	8504	7.40 × 10^-5^	3.34 × 10^-6^	96.1	8.31 × 10^-9^	2.32 × 10^-4^
8.64	8458	7.07 × 10^-5^	2.54 × 10^-6^	97.0	8.05 × 10^-9^	1.54 × 10^-4^
8.70	8529	6.91 × 10^-5^	2.04 × 10^-6^	97.7	7.87 × 10^-9^	1.35 × 10^-4^
8.76	8484	6.65 × 10^-5^	1.67 × 10^-6^	98.1	7.65 × 10^-9^	1.44 × 10^-4^
6.20	8830	9.69 × 10^-5^	8.19 × 10^-5^	15.6	1.70 × 10^-9^	7.51 × 10^-6^
6.59	9879	1.03 × 10^-4^	7.35 × 10^-5^	29.0	3.02 × 10^-9^	2.24 × 10^-5^
7.00	10715	1.14 × 10^-4^	5.89 × 10^-5^	48.8	5.18 × 10^-9^	3.36 × 10^-5^
7.34	11091	1.06 × 10^-4^	4.12 × 10^-5^	61.5	5.85 × 10^-9^	5.96 × 10^-5^
7.56	10071	9.88 × 10^-5^	2.94 × 10^-5^	70.7	6.89 × 10^-9^	6.61 × 10^-5^
7.50	10733	1.02 × 10^-4^	3.59 × 10^-5^	65.3	6.20 × 10^-9^	1.39 × 10^-4^
7.90	9476	8.96 × 10^-5^	1.78 × 10^-5^	80.6	7.58 × 10^-9^	1.93 × 10^-4^
8.13	9062	8.45 × 10^-5^	1.15 × 10^-5^	86.9	8.06 × 10^-9^	1.94 × 10^-4^
8.24	8935	8.04 × 10^-5^	1.19 × 10^-5^	85.7	7.66 × 10^-9^	2.09 × 10^-4^
8.44	8563	7.28 × 10^-5^	4.32 × 10^-6^	94.7	8.00 × 10^-9^	2.70 × 10^-4^
8.67	8495	6.37 × 10^-5^	2.85 × 10^-6^	96.2	7.17 × 10^-9^	2.36 × 10^-4^
8.89	8491	5.66 × 10^-5^	1.32 × 10^-6^	98.5	6.51 × 10^-9^	2.34 × 10^-4^
8.98	8696	5.34 × 10^-5^	1.12 × 10^-6^	98.8	6.02 × 10^-9^	2.06 × 10^-4^

**Table 4:** Solution analyses for strontium sorption to goethite in nitrogen atmosphere, I = 0.1 M NaCl and T = 25°C.

**Final pH**	**Surface Area m^**2 **^L^**-1**^**	**Initial [Sr] M**	**Final [Sr] M**	**%Sr sorbed**	**Γ Sr sorbed Mol m^**-2**^**
Total Sr = 10^-3 ^M, aged for 2 days				± 5%	

6.53	1412	9.20 × 10^-4^	9.44 × 10^-4^	-2.5	-1.65 × 10^-8^
6.79	1435	9.25 × 10^-4^	9.26 × 10^-4^	-0.2	-1.01 × 10^-9^
6.96	1457	9.26 × 10^-4^	9.06 × 10^-4^	2.2	1.37 × 10^-8^
7.26	1565	9.30 × 10^-4^	8.47 × 10^-4^	8.9	5.30 × 10^-8^
7.40	1449	9.27 × 10^-4^	8.07 × 10^-4^	12.9	8.28 × 10^-8^
7.82	1433	9.23 × 10^-4^	6.85 × 10^-4^	25.8	1.66 × 10^-7^
8.30	1478	9.20 × 10^-4^	6.23 × 10^-4^	32.3	2.01 × 10^-7^
8.91	1479	9.16 × 10^-4^	5.27 × 10^-4^	42.5	2.63 × 10^-7^
9.49	1445	9.12 × 10^-4^	3.28 × 10^-4^	64.0	4.04 × 10^-7^
6.23	1431	9.61 × 10^-4^	9.45 × 10^-4^	1.6	1.08 × 10^-8^
6.74	1494	9.66 × 10^-4^	8.98 × 10^-4^	7.0	4.56 × 10^-8^
7.16	1472	9.68 × 10^-4^	9.30 × 10^-4^	3.9	2.58 × 10^-8^
7.47	1472	9.71 × 10^-4^	7.87 × 10^-4^	19.0	1.25 × 10^-7^
8.19	1491	9.76 × 10^-4^	7.67 × 10^-4^	21.5	1.40 × 10^-7^
8.51	1482	9.73 × 10^-4^	7.71 × 10^-4^	20.8	1.37 × 10^-7^
8.88	1481	9.69 × 10^-4^	5.56 × 10^-4^	42.6	2.79 × 10^-7^
9.25	1462	9.63 × 10^-4^	3.29 × 10^-4^	65.8	4.34 × 10^-7^
10.02	1452	9.55 × 10^-4^	9.74 × 10^-5^	89.8	5.91 × 10^-7^

Total Sr = 10^-4 ^M, aged for 2 days				± 5%	

6.43	1570	1.09 × 10^-4^	1.16 × 10^-4^	-5.5	-3.85 × 10^-9^
6.78	1433	1.10 × 10^-4^	1.10 × 10^-4^	-0.6	-4.52 × 10^-10^
7.23	1431	1.10 × 10^-4^	1.13 × 10^-4^	-2.7	-2.09 × 10^-9^
7.49	1442	1.11 × 10^-4^	1.10 × 10^-4^	1.0	7.44 × 10^-10^
7.78	1602	1.11 × 10^-4^	1.00 × 10^-4^	9.6	6.63 × 10^-9^
8.19	1555	1.11 × 10^-4^	8.76 × 10^-5^	20.8	1.48 × 10^-8^
8.89	1511	1.10 × 10^-4^	4.53 × 10^-5^	58.7	4.26 × 10^-8^
9.38	1532	1.10 × 10^-4^	3.44 × 10^-5^	68.6	4.90 × 10^-8^
9.9	1540	1.09 × 10^-4^	1.88 × 10^-5^	82.7	5.85 × 10^-8^

Total Sr = 10^-5 ^M, aged for 2 days				± 5%	

6.43	1540	1.08 × 10^-5^	1.06 × 10^-5^	1.4	9.51 × 10^-11^
6.74	1534	1.08 × 10^-5^	1.05 × 10^-5^	3.1	2.19 × 10^-10^
7.30	1501	1.09 × 10^-5^	1.07 × 10^-5^	1.8	1.29 × 10^-10^
7.73	1423	1.09 × 10^-5^	9.67 × 10^-6^	11.2	8.54 × 10^-10^
8.05	1463	1.09 × 10^-5^	9.31 × 10^-6^	14.6	1.08 × 10^-9^
8.42	1503	1.09 × 10^-5^	7.79 × 10^-6^	28.4	2.05 × 10^-9^
9.22	1502	1.08 × 10^-5^	3.88 × 10^-6^	64.2	4.62 × 10^-9^
9.79	1481	1.08 × 10^-5^	1.61 × 10^-6^	85.0	6.19 × 10^-9^
10.21	1416	1.07 × 10^-5^	7.34 × 10^-7^	93.2	7.06 × 10^-9^

**Table 5:** Solution analyses for strontium sorption to goethite in air, I = 0.1 M NaCl and T = 25°C.

**Final pH**	**Surface Area m^**2 **^L^**-1**^**	**Initial [Sr] M**	**Final [Sr] M**	**%Sr sorbed**	**Γ Sr sorbed Mol m^**-2**^**	**Total Dissolved Carbonate M**
Total Sr = 10^-4 ^M aged for 2 days				± 5%		

6.43	1299	1.10 × 10^-4^	1.06 × 10^-4^	3.9	3.34 × 10^-9^	not measured
6.85	1304	1.10 × 10^-4^	1.07 × 10^-4^	2.5	2.13 × 10^-9^	not measured
7.23	1307	1.10 × 10^-4^	1.08 × 10^-4^	1.8	1.49 × 10^-9^	not measured
7.62	1311	1.11 × 10^-4^	1.02 × 10^-4^	8.4	7.13 × 10^-9^	not measured
7.97	1309	1.11 × 10^-4^	1.03 × 10^-4^	6.8	5.71 × 10^-9^	not measured
8.29	1305	1.1 × 10^-4^	1.17 × 10^-4^	-5.8	-4.92 × 10^-9^	not measured
8.96	1304	1.10 × 10^-4^	6.18 × 10^-4^	43.7	3.68 × 10^-8^	not measured
9.28	1297	1.09 × 10^-4^	4.11 × 10^-4^	62.4	5.26 × 10^-8^	not measured
9.97	1278	1.08 × 10^-4^	2.30 × 10^-5^	78.8	6.68 × 10^-8^	not measured

Total Sr = 10^-4 ^M aged for 14 days				± 5%		

6.52	1478	1.11 × 10^-4^	1.09 × 10^-4^	1.9	1.43 × 10^-9^	1.47 × 10^-4^
7.03	1513	1.11 × 10^-4^	1.06 × 10^-4^	5.0	3.69 × 10^-9^	2.10 × 10^-4^
7.50	1523	1.12 × 10^-4^	1.01 × 10^-4^	9.5	6.97 × 10^-9^	3.76 × 10^-4^
8.12	1491	1.11 × 10^-4^	8.20 × 10^-5^	26.4	1.97 × 10^-8^	5.29 × 10^-4^
8.18	1507	1.11 × 10^-4^	6.84 × 10^-5^	38.4	2.83 × 10^-8^	6.38 × 10^-4^
9.19	1478	1.11 × 10^-4^	4.20 × 10^-5^	62.0	4.64 × 10^-8^	7.22 × 10^-4^
9.84	1484	1.10 × 10^-4^	2.17 × 10^-5^	80.2	5.93 × 10^-8^	8.93 × 10^-4^
10.59	1453	1.09 × 10^-4^	7.54 × 10^-6^	93.1	6.96 × 10^-8^	9.97 × 10^-4^
11.32	1416	1.06 × 10^-4^	2.15 × 10^-6^	98.0	7.34 × 10^-8^	1.07 × 10^-3^

Total Sr = 10^-5 ^M aged for 2 days				± 5%		

6.89	1301	1.17 × 10^-5^	9.81 × 10^-6^	16.4	1.48 × 10^-9^	not measured
7.18	1310	1.18 × 10^-5^	1.00 × 10^-5^	15.0	1.35 × 10^-9^	not measured
7.51	1309	1.18 × 10^-5^	9.49 × 10^-6^	19.6	1.77 × 10^-9^	not measured
7.86	1308	1.18 × 10^-5^	8.64 × 10^-6^	26.7	2.41 × 10^-9^	not measured
8.18	1304	1.18 × 10^-5^	7.36 × 10^-6^	37.4	3.37 × 10^-9^	not measured
8.76	1300	1.17 × 10^-5^	4.51 × 10^-6^	61.4	5.52 × 10^-9^	not measured
9.44	1293	1.16 × 10^-5^	3.45 × 10^-6^	70.4	6.34 × 10^-9^	not measured
10.05	1285	1.15 × 10^-5^	7.57 × 10^-7^	93.4	8.40 × 10^-9^	not measured

Total Sr = 10^-5 ^M aged for 14 days				± 5%		

6.60	1484	1.30 × 10^-5^	1.25 × 10^-5^	3.1	2.74 × 10^-10^	1.04 × 10^-4^
7.17	1482	1.30 × 10^-5^	1.27 × 10^-5^	2.4	2.09 × 10^-10^	2.08 × 10^-4^
8.36	1441	1.30 × 10^-5^	7.73 × 10^-6^	40.6	3.66 × 10^-9^	5.83 × 10^-4^
9.36	1488	1.29 × 10^-5^	2.06 × 10^-6^	84.1	7.30 × 10^-9^	8.14 × 10^-4^
9.92	1491	1.28 × 10^-5^	6.79 × 10^-7^	94.7	8.15 × 10^-9^	9.33 × 10^-4^
10.55	1485	1.27 × 10^-5^	1.33 × 10^-7^	99.0	8.46 × 10^-9^	1.08 × 10^-3^

Total Sr = 1.3 × 10^-6 ^M aged for 2 days				(± 12%)		

6.28	1296	1.28 × 10^-6^	1.19 × 10^-6^	7.1	7.03E × 10^-11^	not measured
6.58	1302	1.29 × 10^-6^	1.31 × 10^-6^	-1.5	-1.50 × 10^-11^	not measured
7.08	1306	1.29 × 10^-6^	1.11 × 10^-6^	14.1	1.39 × 10^-10^	not measured
7.38	1131	1.27 × 10^-6^	1.11 × 10^-6^	12.6	1.41 × 10^-10^	not measured
7.65	1090	1.26 × 10^-6^	9.95 × 10^-7^	21.1	2.43 × 10^-10^	not measured
8.09	1057	1.25 × 10^-6^	7.21 × 10^-7^	42.4	5.02 × 10^-10^	not measured
8.78	1055	1.25 × 10^-6^	3.55 × 10^-7^	71.5	8.45 × 10^-10^	not measured
9.49	970	1.23 × 10^-6^	1.26 × 10^-7^	89.8	1.14 × 10^-9^	not measured
10.05	884	1.21 × 10^-6^	3.44 × 10^-8^	97.2	1.33 × 10^-9^	not measured

**Table 6:** Solution analyses for strontium sorption to goethite in air, I = 0.1 M NaNO_3 _and T = 25°C.

**Final pH**	**Surface Area m^**2 **^L^**-1**^**	**Initial [Sr] M**	**Final [Sr] M**	**%Sr sorbed**	**Γ Sr sorbed Mol m^**-2**^**	**Total Dissolved Carbonate M**
Total Sr = 10^-4^M aged for 2 days				± 5%		

3.98	266	9.61 × 10^-5^	9.44 × 10^-5^	1.8	6.60 × 10^-9^	not measured
4.89	271	9.79 × 10^-5^	9.79 × 10^-5^	0.1	2.00 × 10^-10^	not measured
6.11	269	9.74 × 10^-5^	9.59 × 10^-5^	1.5	5.33 × 10^-9^	not measured
7.13	265	9.58 × 10^-5^	1.01 × 10^-4^	-5.1	-1.86 × 10^-8^	not measured
7.95	264	9.55 × 10^-5^	9.11 × 10^-5^	4.6	1.67 × 10^-8^	not measured
8.85	263	9.50 × 10^-5^	7.97 × 10^-5^	16.1	5.82 × 10^-8^	not measured
9.91	261	9.44 × 10^-5^	4.61 × 10^-5^	51.2	1.85 × 10^-7^	not measured

Total Sr = 10^-5 ^M aged for 2 days				± 5%		

3.99	275	1.01 × 10^-5^	9.86 × 10^-6^	2.0	7.19 × 10^-10^	not measured
5.09	275	1.00 × 10^-5^	1.02 × 10^-5^	-2.2	-8.14 × 10^-10^	not measured
6.22	274	1.00 × 10^-5^	1.01 × 10^-5^	-0.8	-2.76 × 10^-10^	not measured
7.29	273	9.99 × 10^-6^	9.35 × 10^-6^	6.4	2.34 × 10^-9^	not measured
8.15	277	1.01 × 10^-5^	7.26 × 10^-6^	28.2	1.03 × 10^-8^	not measured
8.92	275	1.01 × 10^-5^	4.54 × 10^-6^	54.8	2.00 × 10^-8^	not measured
9.95	271	9.91 × 10^-6^	1.60 × 10^-6^	83.9	3.06 × 10^-8^	not measured

Total Sr = 1.1 × 10^-6 ^M aged for 2 days				(± 7%)		

4.04	273	1.10 × 10^-6^	9.82 × 10^-7^	10.4	4.17 × 10^-10^	not measured
4.97	274	1.10 × 10^-6^	9.93 × 10^-7^	9.6	3.83 × 10^-10^	not measured
6.11	273	1.09 × 10^-6^	1.02 × 10^-6^	7.1	2.84 × 10^-10^	not measured
7.22	275	1.10 × 10^-6^	1.04 × 10^-6^	5.6	2.25 × 10^-10^	not measured
7.95	271	1.09 × 10^-6^	9.59 × 10^-7^	11.8	4.73 × 10^-10^	not measured
8.95	273	1.09 × 10^-6^	5.14 × 10^-7^	53.0	2.13 × 10^-9^	not measured
9.94	270	1.08 × 10^-6^	1.14 × 10^-7^	89.5	3.59 × 10^-9^	not measured

### EXAFS sorption samples

Strontium sorption to amorphous silica experiments from pH 8 to 10 with dissolved CO_2 _and initial strontium concentrations of 10^-3 ^M were analyzed with EXAFS. After reaction and centrifugation, supernatant liquids were removed and sorption samples were loaded as wet pastes into teflon sample holders with Kapton windows just prior to XAS analysis. For sorption samples collected at cryogenic temperatures, wet samples were quenched by immersion in liquid nitrogen and then placed in a helium cryostat in the beamline hutch.

For strontium sorption samples, EXAFS spectra were collected on wiggler beamline IV-3 at the Stanford Synchrotron Radiation Laboratory (SSRL). The incident beam was detuned to 50–70% of the maximum incoming intensity to reject higher-order harmonic reflections. The mid-point of the absorption edge of SrCO_3_(s) reference compound (set to 16105 eV) was used for energy calibration. Spectra were collected in fluorescence mode using a 13-element germanium array detector. For each sorption sample, 20–40 scans were collected to achieve an adequate signal.

Spectra in the EXAFS region were analyzed with the program EXAFSPAK [30]. Reference phase shift and amplitude functions used in non-linear least-squares fitting of experimental spectra were calculated using the *ab initio *program FEFF6 [31-33]. In non-linear least-squares fits of the sorption sample spectra, bond distance (R), backscatterer number (N), and the disorder or Debye-Waller term (*σ*^2^) were treated as adjustable parameters. The difference between theoretical and experimental threshold energies (ΔE_0_) was treated as a single adjustable parameter for all Sr-backscatterer shells [34]. Least-squares fits were performed on both filtered spectra of individual peaks in the radial structure functions (RSF) and on normalized *χ*(k) spectra with no significant differences in fit results. Detailed analyses of crystalline and hydrated strontium reference compounds and strontium in aqueous solution at ambient and cryogenic temperatures are given in our previous study [25]. These references allowed us to constrain adjustable EXAFS fitting parameters and to estimate errors in fit parameters based on empirical analysis (rather than using only the statistical errors derived from the least-squares fit). Our previous study [25], showed that anharmonic vibrational disorder of oxygen-ligated strontium compounds can be neglected because the third cummulate term (C3) of the EXAFS phase shift function is generally not significant above the error in fitted EXAFS distances (i.e., R ± 0.02 Å with and without C3). Also, we showed that rapid quenching of sorption samples for data collection at low temperature does not appear to introduce any new features into EXAFS spectra when compared with room temperature spectra. Based on our previous strontium EXAFS analyses, S_0_^2 ^was fixed at 0.92 and estimated empirical errors in fit parameters for first-shell Sr-O analysis are: R ± 0.02 Å; N ± 1 for N in the range of 6–12; *σ*^2 ^± 25% [25,26].

### Geochemical calculations

GEOSURF [35] and FITEQL [36] were used to fit specific surface complexation reactions to the experimental data. GEOSURF is tied to a thermodynamic database that automatically accounts for aqueous speciation and ionic strength during the sorption simulation. We used GEOSURF to fit strontium sorption to amorphous silica and goethite in carbonate-free suspensions and to amorphous silica in the presence of dissolved carbonate because SrCO_3_(aq) is negligible and dissolved carbonate is not known to sorb to amorphous silica to the best of our knowledge. We used FITEQL to fit strontium sorption data to goethite in the presence of dissolved carbonate to account for the significant carbonate sorption to the goethite surface because FITEQL allows the input of measured carbonate concentrations at each titration point for the speciation calculation. The pH dependence of measured dissolved carbonate concentrations in goethite suspensions was used to estimate dissolved carbonate concentrations when modeling strontium sorption for those experiments in which dissolved carbonate was not measured. The extended-Debye Hückel equation was used to correct for ionic strength effects. Aqueous equilibrium constants used in the calculations are listed in Table 7[37].

**Table 7:** Thermodynamic equilibrium constants used to account for aqueous speciation for strontium sorption model [37].

Mass balance reactions	log K
H_2_O = H^+ ^+ OH^-^	-14
HCO_3_^- ^= CO_3_^-- ^+ H^+^	-10.33
CO_2_(aq) + H_2_O = 2 H^+ ^+ CO_3_^--^	-16.67
NaCl(aq) = Na^+ ^+ Cl^-^	0.78
SrCl^+ ^= Sr^++ ^+ Cl^-^	0.25
SrCO_3_(aq) = Sr^++ ^+ CO_3_^--^	2.87

## Results

### EXAFS analysis

**Figure 1:**
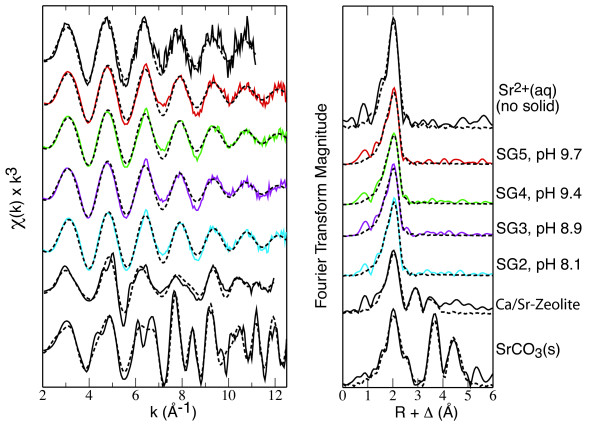
Normalized EXAFS spectra and corresponding radial structural functions for strontium sorbed to amorphous silica and for Sr^2+^(aq), strontianite (SrCO_3_(s)), and a Ca/Sr zeolite models. Solid lines are the data and dashed lines are fits to the data. For all sorption samples collected at low temperature, there is only a single shell of oxygen backscatterers indicated by one peak in the radial structural functions compared to multiple peaks for the model compounds reflecting Sr-Sr, Sr-C and Sr-Si/Al backscatters.

Absorption spectra were collected for samples of strontium sorbed to amorphous silica from solutions of 10^-3 ^M SrCl_2 _and 0.1 M NaCl with dissolved CO_2 _from pH 8 to 10. Normalized *χ*(k) EXAFS spectra and Fourier transforms of the spectra are shown in Figure 1. Numerical fit results are given in Table 8. For all sorption samples collected at low temperature, there is only a single shell of oxygen backscatterers with a Sr-O distance of 2.60 ± 0.02 Å and a coordination number of 10 ± 1. Compared to a spectrum of strontium sorbed to silica gel collected at room temperature reported in our previous study [26], the fitted Sr-O distance at room temperature is slightly shorter (2.57 ± 0.02 Å) than that derived for low-temperature spectra. This small distance contraction was also noted for room- and low-temperature spectra of strontium sorbed to kaolinite [26] and probably results from a small anharmonic effect [25]. For strontium sorption on both silica gel and kaolinite, the fitted Sr-O distance is slightly shorter than the Sr-O distance (2.65 ± 0.02 Å) determined for aqueous Sr^2+ ^in a 10^-3 ^M SrCl_2 _solution [25].

**Table 8:** EXAFS fit results for strontium sorbed to amorphous silica and goethite (R, N, *σ*^2 ^and ΔE_0 _adjusted in fits).

Electrolyte	pH	T(K)	Sr-Z^*a*^	R(Å)	N	*σ*^2^(Å^2^)	ΔE_0_(eV)
Amorphous Silica, Sr_total _= 10^-3 ^M							

0.1 M Na Cl	8.1	20	Sr-O	2.60	10.0	0.0081	-4.9
0.1 M Na Cl	8.9	20	Sr-O	2.60	10.1	0.0087	-5.4
0.1 M Na Cl	9.4	25	Sr-O	2.60	10.6	0.0094	-6.1
0.1 M Na Cl	9.7	16	Sr-O	2.60	10.3	0.0086	-5.9

Goethite^*c*^, Sr_total _= 10^-3 ^M							

0.1 M Na Cl	8.5	20	Sr-O	2.63	10.1	0.0084	-6.6
			Sr-C	3.05	3.8	0.0018	
			Sr-Sr	4.14	3.4	0.0021	
			Sr-Sr	4.28	1.4	0.0005	
			Sr-Sr	4.90	3.3	0.0031	
0.1 M Na Cl	8.7	RT	Sr-O	2.58	8.3	0.0126	-5.6
0.1 M Na Cl	9.3	15	Sr-O	2.61	9.8	0.0081	-5.0
0.1 M NaNO_3_	9.9	12	Sr-O	2.60	9.1	0.0077	-6.9

^*a*^Z = backscattering atom.

^*b*^Scale factor (S_0_^2^) = 0.92

^*c*^Goethite samples and analyses are from Sahai et al. [26]

**Figure 2:**
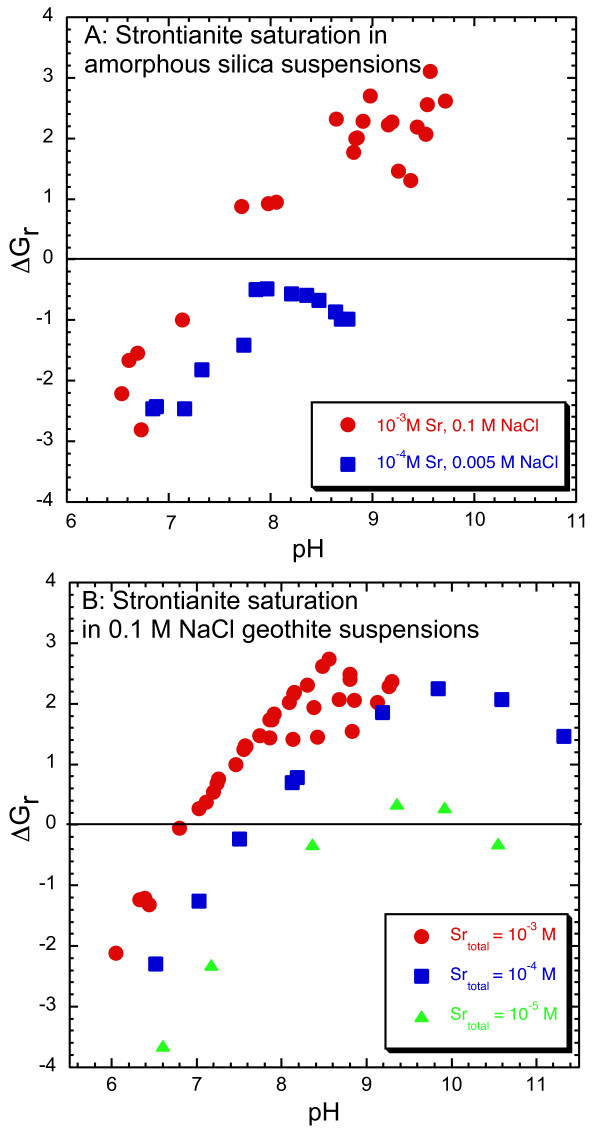
ΔG_r _for final solution compositions with respect to strontianite (SrCO_3_) in which both dissolved strontium and carbonate were measured from amorphous silica (A) and goethite (B) suspensions. Solutions are supersaturated when ΔG_r _is > 0 and undersaturated when ΔG_r _< 0.

Comparison of EXAFS sorption spectra indicates no change in strontium coordination with increasing pH and sorption. There is no evidence for silica backscatterers from the substrate, nor is there evidence for carbon or strontium backscatterers indicative of strontianite precipitation or other multi-nuclear sorption complexes. Evidence for scattering from atoms beyond the first coordination shell would be seen in multiple sine-wave oscillations in normalized spectra (i.e., "beat" patterns or shoulders on primary sine waves). Figure 1 compares the strontium sorption spectra to reference spectra for crystalline strontium carbonate (SrCO_3_(s)) and strontium in the calcium zeolite mineral heulandite (≈ 4500 ppm strontium substitution in the calcium site). In the calcium site in heulandite, strontium is eight-coordinated by oxygen, with three ligands of framework oxygen atoms from the mineral surface and five ligands of water extending into the zeolite channel. As shown here and in our previous study of strontium in zeolites [25], scattering from aluminum or silicon atoms in the zeolite framework is apparent in spectra up to a distance of 4.15 Å from central strontium because of direct bonding to the zeolite framework. Thus, strontium in zeolites is a good analog structure for inner-sphere complexation of strontium on silica gel, if it occurs. Although aluminum and silicon are relatively light backscatterers, they are easily identified as backscatterers in zeolite when strontium is partially dehydrated and bonds to the framework structure. Likewise, precipitation of strontianite is readily observed by backscattering from carbon and strontium atoms at distances of ≈ 4 Å or less, but is not seen in the sorption sample spectra, even for samples in which reacting solutions were supersaturated with respect to strontianite (Figure 2a). In our previous study of strontium reference compounds [25], we found that backscatterer atoms beyond the first oxygen coordination shell were apparent in normalized spectra when fitted values of *σ*^2 ^(the Debye-Waller disorder parameter) were below ≈ 0.025 Å ^2 ^(for N < 12 and R > 3 Å). We did not collect EXAFS spectra on samples of strontium sorbed to amorphous silica in the absence of CO_2 _because the bulk sorption behavior was the same with CO_2 _present. Nor did we collect EXAFS spectra on strontium sorption in 5 × 10^-3 ^M NaCl solutions.

**Figure 3:**
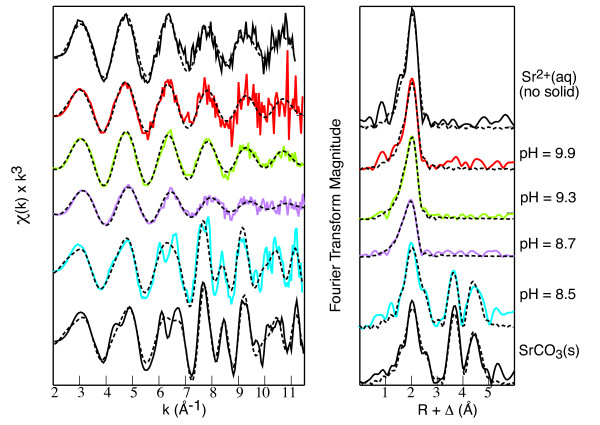
Normalized EXAFS spectra and corresponding radial structural functions for strontium sorbed to goethite and for Sr^2+^(aq) and SrCO_3_(s) models (reproduced from Sahai et al., 2000). Solid lines are the data and dashed lines are fits to the data. At pH 8.5 multiple peaks in the radial structural functions reflect Sr-O, Sr-C, and Sr-Sr backscatters that match SrCO_3 _(s). At higher pH, there is only a single shell of oxygen backscatterers indicated by one peak in the radial structural functions.

Figure 3 and Table 8 reproduce EXAFS spectra on strontium sorption to goethite with dissolved carbonate [26] to show changes in bonding at the surface as a function of solution pH. These results show that strontium forms a surface precipitate at pH 8.5, but not at higher pH where the solutions were more supersaturated with respect to strontianite for total strontium concentrations of 10^-3 ^M (Figure 2b). For solutions with pH above 8.5 only Sr-O backscatters were detected indicating that strontium retains all or part of its hydration sphere when sorbed to the surface. This behavior was attributed to a maximum sorption of carbonate on goethite near pH 8.5 that nucleated a SrCO_3_-type surface precipitate, and decreasing carbonate sorption at higher pH that resulted in formation of hydrated Sr surface complexes [26].

### Macroscopic sorption experiments

#### Amorphous silica

**Figure 4:**
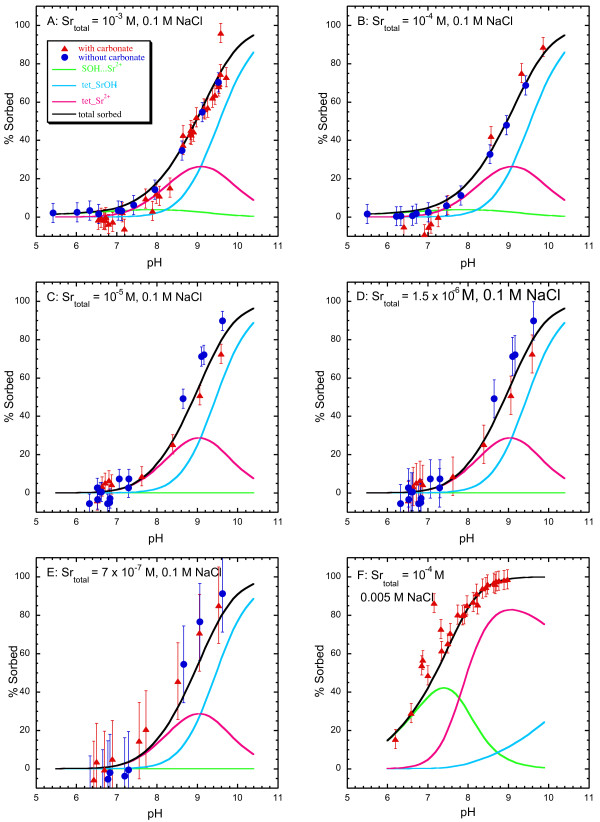
Comparison of experimental and model results for strontium sorption to amorphous silica in 7 × 10^-7 ^to 10^-3 ^M SrCl_2 _and 0.1 M NaCl solutions with and without dissolved carbonate (A-E), and in 10^-4 ^M SrCl_2 _and 0.005 M NaCl solutions with dissolved carbonate (F). Symbols are the experimental data and solid lines are fits to the data.

The pH dependence of strontium sorption to amorphous silica in ~7 × 10^-7 ^to 10^-3 ^M SrCl_2 _and 0.1 M NaCl solutions with and without dissolved carbonate is shown in Figure 4 and Tables 1 and 2. Strontium sorption was near zero below pH 7.0 and increased with pH to about 80 % of the initial strontium concentration at pH 9.5 for all experiments. There was no measurable effect of dissolved CO_2 _on strontium sorption as shown by the similar pH dependence of sorption in suspensions with and without dissolved carbonate. Carbonate sorption to amorphous silica was not measured. We infer that strontium carbonate precipitation was negligible because it was not detected by EXAFS, even though final solution compositions for experiments with total strontium concentrations of 10^-3 ^and 10^-4 ^M are supersaturated or approach saturation with respect to strontianite (SrCO_3_) with increasing pH (Figure 2).

Strontium sorption to amorphous silica in 10^-4 ^M SrCl_2 _and 5 × 10^-3 ^M NaCl solutions prepared in the presence of dissolved carbonate is shown in Figure 4F and Table 3. These experiments were conducted to investigate the effect ionic strength on sorption. The strontium sorption increases at lower ionic strength as shown by a shift in the midpoint of the sorption edge (50% sorbed Sr) from pH 9.0 in 0.1 M NaCl solutions (Fig. 4B) to pH 7.2 in 5 × 10^-3 ^M NaCl solutions (Fig. 4F). A similar shift in the strontium sorption edge has been observed for strontium sorbed to amorphous silica in 0.1 M and 0.01 M NaNO_3_, NaCl, and NaClO_4 _background electrolytes [28].

#### Goethite

**Figure 5:**
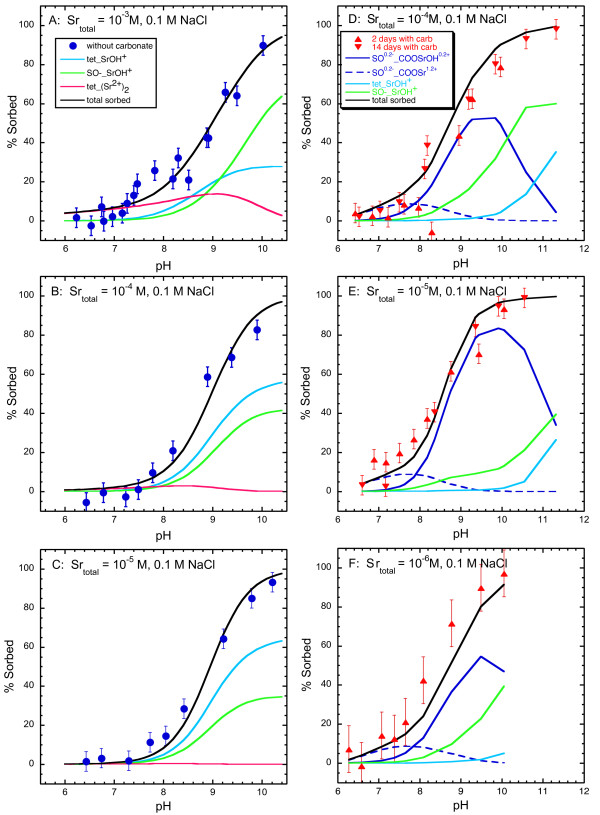
Comparison of experimental and model results for strontium sorption to goethite in 10^-5 ^to 10^-3 ^M SrCl_2 _and 0.1 M NaCl solutions without dissolved carbonate (A-C) and in 10^-6 ^to 10^-4 ^M SrCl_2 _and 0.1 M NaCl solutions with dissolved carbonate (D-F). Symbols are the experimental data and solid lines are fits to the data.

The pH dependence of strontium sorption to goethite in 10^-5 ^to 10^-3 ^M SrCl_2 _and 0.1 M NaCl solutions without dissolved carbonate are shown in Figure 5(A–C) and Table 4. Similar to amorphous silica, strontium sorption to goethite has a broad pH sorption edge from pH 7 to 10. Enhanced strontium sorption to goethite between pH 7 and 8 with total strontium concentrations of 10^-3 ^M was observed compared to lower total strontium concentrations of 10^-4 ^and 10^-5 ^M. The enhanced sorption at Sr = 10^-3 ^M was reproduced in duplicate experiments and does not appear to be an experimental artifact.

**Figure 6:**
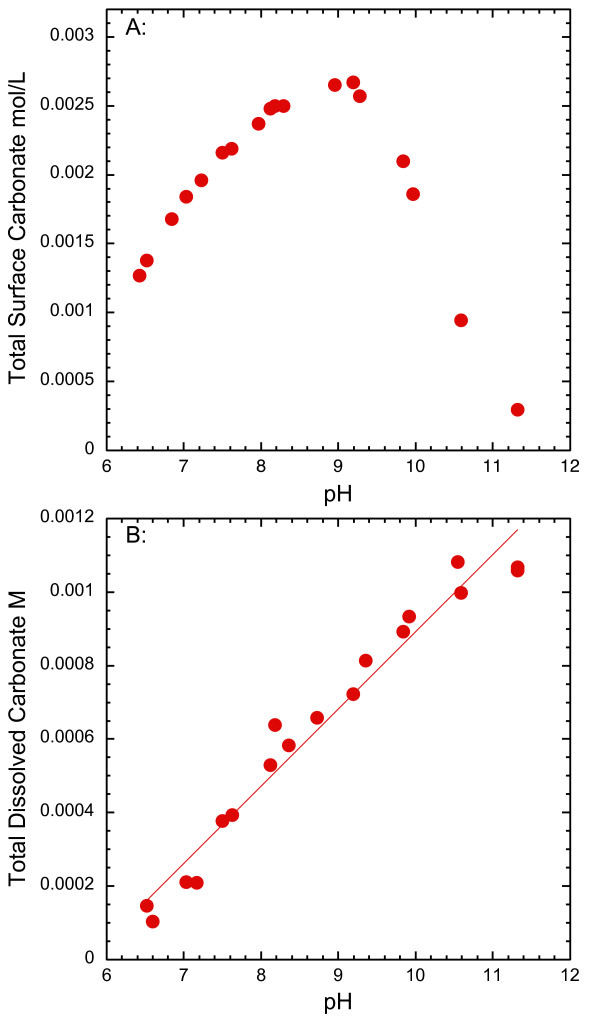
Total carbonate predicted at the goethite surface versus pH (A) in 0.1 M NaCl suspensions using measured carbonate concentrations (B) and strontium carbonate complexation model (Table 9).

The pH dependence of strontium sorption to goethite with dissolved carbonate in 10^-6 ^to 10^-4 ^M Sr in 0.1 M NaCl (aged for 2 and 14 days) and 0.1 M NaNO_3 _(aged for 2 days) solutions is shown in Figures 5(D–F) and 6 and Tables 5 and 6. Similar to strontium sorption in carbonate-free systems, strontium sorption exhibits a broad sorption edge from pH 7 to 10. In the presence of dissolved carbonate, strontium carbonate precipitation has been observed at high total strontium concentrations [26] and may form strontium carbonate surface complexes because dissolved carbonate is known to sorb to goethite [38-42]. In our studies, strontium carbonate precipitation at the goethite surface appears to be negligible for Sr of 10^-6 ^to 10^-4 ^M. We assume that surface precipitation of SrCO_3 _(s) is minimal in these experiments because it was not detected in an EXAFS spectra from a strontium sorption sample prepared in the presence of atmospheric CO_2 _with pH = 9.9 and total Sr ~ 10^-4 ^M where final solutions are highly supersaturated with respect to strontianite (Figures 2 and 3). It is possible that strontium carbonate precipitates at the goethite surface at lower pH even though the extent of supersaturation is less, because surface precipitates have been observed at pH 8.5 but not at higher pH in solutions with total Sr = 10^-3 ^M [26].

## Discussion

The strontium surface complexation model for amorphous silica and goethite presented here builds on a triple layer alkaline earth surface complexation model developed for oxides and hydroxides [16]. The model defines a limited number of surface complexation reactions that describe alkaline earth sorption over a wide range of ionic strength, pH, surface coverage, and oxide type by collectively fitting experimental data which investigated a limited set of these variables [43-54]. We model strontium sorption to amorphous silica and goethite using the same set of surface species to contribute to an internally consistent model for alkaline earth sorption that can be used for a wide range of solution and substrate compositions. Alkaline earth sorption is modeled primarily as a series of surfaces complexes placed on the *β*- or diffuse planes to account for the broad sorption edge, the dependence of sorption on ionic strength and available spectroscopic data. Although these complexes are not strictly identified as outer-sphere complexes by Sverjensky [16], their placement in the *β*- or diffuse planes is consistent with EXAFS analysis, which show that sorbed strontium remains hydrated at amorphous silica and goethite surfaces (this study, [26,28]). The stoichiometry of surface complexes is further constrained by requiring the formation of some tetradentate complexes because strontium was found to bond to four surface oxygens on rutile with X-ray standing wave spectroscopy [27]. Although the exact stoichiometry could not be identified with spectroscopy, Sverjensky [16] found that the surface species (>SOH)_2_(>SiO^-^)_2__MOH^+ ^or (>SOH)_2_(>SiO^-^)_2__M^2+^, where M stands for any alkaline earth, captured the broad pH dependence for much of the alkaline earth sorption.

Strontium surface complexation reactions were fit to the experimental data using equilibrium constants from Sverjensky [15] to account for surface protonation and sorption of the background electrolyte. All equilibrium constants were adjusted in accordance with the site-occupancy standard state [14]. Table 9 reports the total number of sites N_s_, solid concentration C_s_, surface area SA, and equilibrium constants normalized to 1 M standard state (K^0^) used in the fitting programs and the site-occupancy standard state (K^*θ*^) used in the Born solvation and crystal-chemistry analysis. The site-occupancy standard state allows sorption experiments to be compared with one another independent of surface area, site density, and solid concentration. All complexes are mononuclear unless stated otherwise. The fitted constants were found to be accurate within an uncertainty of ± 0.3 log K [16].

**Table 9:** Equilibrium constants for alkaline earth sorption, where Log K^0 ^is the equilibrium constant referenced to a 1 M standard state, Log K^θ ^is the equilibrium constant referenced to a site-occupancy standard state, N_s _is the site density (# nm^-2^), A_s _is the surface area (m^2^g^-1^), and C_s _is the solid concentration (g L^-1^). The site-occupancy standard state assumes reference N_s _= 10 sites nm^-2 ^and A_s _= 10 m^2^g^-1^.

**Solid**	**Log K^0^**	**Surface Complexation Reaction**	**N_s_**	**A_s_**	**C_s_**	**Log K^θ^**
^a^Amorphous silca	-2.5	>SOH + H^+ ^= >SOH_2_^+^	4.6	277	40	-1.4
	-5.9	>SOH = >SO^- ^+ H^+^	4.6	277	40	^d^7.0
	-1.4	>SOH + H^+ ^+ Cl^- ^= >SOH_2_^+^_Cl^-^	4.6	277	40	1.1
	-7.2	>SOH + Na^+ ^= >SO^-^_Na^+ ^+ H^+^	4.6	277	40	^d^0.9
	-1.5	>SOH + Sr^2+ ^= >SOH...Sr^2+^	4.6	277	40	-0.4
	-10.0	4>SOH + Sr^2+ ^= (>SOH)_2_(>SO^-^)_2__Sr^2+ ^+ 2H^+^	4.6	277	40	^d^19.2
	-16.2	4>SOH + Sr^2+ ^+ H_2_O = (>SOH)_2_(>SO^-^)_2__SrOH^+ ^+ 3H^+^	4.6	277	40	^d ^27.0

^b^Goethite	5.6	>SOH + H^+ ^= >SOH_2_^+^	16.4	37.7	40	6.4
	-11.2	>SOH = >SO^- ^+ H^+^	16.4	37.7	40	^d ^12.0
	8.9	>SOH + H^+ ^+ Cl^- ^= >SOH_2_^+^_Cl^-^	16.4	37.7	40	3.3
	-9.3	>SOH + Na^+ ^= >SO^-^_Na^+ ^+ H^+^	16.4	37.7	40	^d^3.5
	-16.6	>SOH + Sr^2+ ^+ H_2_O = >SO^-^_SrOH^+ ^+ 2H^+^	16.4	37.7	40	^d^10.2
	-20.7	4>SOH + Sr^2+ ^+ H2O = (>SOH)_2_(>SO^-^)_2__SrOH^+ ^+ 3H^+^	16.4	37.7	40	^d^31.2
	-10.0	4>SOH + 2Sr^2+ ^= (>SOH)_2_(>SO^-^)_2__Sr_2_^4+ ^+ 2H^+^	16.4	37.7	40	^d^15.9
	13.8	>SOH + H^+ ^+ CO_3_^2- ^= >SO^-2^_COO^-8 ^+ H_2_O	16.4	37.7	40	13.0
	13.2	>SOH + H^+ ^+ Na^+ ^+ CO_3_^2- ^= >SOCOONa + H_2_O	16.4	37.7	40	12.4
	18.6	>SOH + 2H^+ ^+ CO_3_^2- ^= >SOCOOH + H_2_O	16.4	37.7	40	17.8
	6.5	>SOH + CO_3_^2- ^+ Sr^2+ ^= >SO^0.2-^_COOSrOH^0.2+^	16.4	37.7	40	5.8
	12.8	>SOH + H^+ ^+ CO_3_^2- ^+ Sr^2+ ^= >SO^0.2-^_COOSr^1.2+ ^+ H_2_O	16.4	37.7	40	12.0

^c^Goethite	5.7	>SOH + H^+ ^= >SOH_2_^+^	16.4	27.7	10	6.4
	-11.3	>SOH = >SO^- ^+ H^+^	16.4	27.7	10	^d ^12.0
	9.1	>SOH + H^+ ^+ Cl^- ^= >SOH_2_^+^_NO_3_^-^	16.4	27.7	10	3.3
	-9.1	>SOH + Na^+ ^= >SO^-^_Na^+ ^+ H^+^	16.4	27.7	10	^d^3.5
	-16.5	>SOH + Sr^2+ ^+ H_2_O = >SO^-^_SrOH^+ ^+ 2H^+^	16.4	27.7	10	^d^10.4
	-18.4	4>SOH + Sr^2+ ^+ H_2_O = (>SOH)_2_(>SO^-^)_2__SrOH^+ ^+ 3H^+^	16.4	27.7	10	^d^31.3
	13.6	>SOH + H^+ ^+ CO_3_^2- ^= >SO^-.2^_COO^-.8 ^+ H_2_O	16.4	27.7	10	13.0
	13.0	>SOH + H^+ ^+ Na^+ ^+ CO_3_^2- ^= >SOCOONa + H_2_O	16.4	27.7	10	12.4
	18.4	>SOH + 2H^+ ^+ CO_3_^2- ^= >SOCOOH + H_2_O	16.4	27.7	10	17.8
	6.4	>SOH + CO_3_^2- ^+ Sr^2+ ^= >SO^0.2-^_COOSrOH^0.2+^	16.4	27.7	10	5.8
	12.7	>SOH + H^+ ^+ CO_3_^2- ^+ Sr^2+ ^= >SO^0.2-^_COOSr^1.2 ^+ H_2_O	16.4	27.7	10	12.0

a. Experiments conducted with and without dissolved carbonate in NaCl solutions, C_1 _= 105 μF cm^-2 ^and C_2 _= 0.2 μF cm^-2^, C_1 _was predicted for amorphous silica from Equation 82 in Sverjensky [16].

b. Experiments conducted with dissolved carbonate in 0.1 M NaCl solutions, C_1 _= 97 μF cm^-2 ^and C_2 _= 0.2 μF cm^-2^. Mid-range value for C_1 _was used in goethite because there is no clear explanation for the wide range of reported values [16].

c. Experiments conducted with dissolved carbonate in 0.1 M NaNO_3 _solutions, C_1 _= 97 μF cm^-2 ^and C_2 _= 0.2 μF cm^-2^. Mid-range value for C_1 _was used in goethite because there is no clear explanation for the wide range of reported values [16].

d. Mass balance reactions for log K^θ ^after Sverjensky [16]. All other mass balance reactions are the same for log K^θ ^and log K^0^.

### Strontium sorption to amorphous silica

The fitted and experimental results are shown in Figure 4 for the sorption of strontium to amorphous silica in solutions with total Sr ranging from ~7 × 10^-7 ^to 10^-3 ^M, 0.1 M NaCl, and with and without dissolved carbonate, and for one experiment with total Sr = 10^-4 ^M, 0.005 M NaCl, and dissolved carbonate. Strontium sorption to amorphous silica in the presence and absence of dissolved carbonate can be described with two tetradentate strontium complexes on the *β*-plane and one monodentate strontium complex on the diffuse plane (Table 9):

4 >SOH + Sr^2+ ^= (>SOH)_2_(>SO-)_2__ Sr^2+ ^+ 2H^+^

4 >SOH + Sr^2+ ^+H_2_O = (>SOH)_2_(>SO-)_2__ SrOH^+ ^+ 3H^+^

>SOH + Sr^2+ ^= >SOH...Sr^2+^

In 0.1 M NaCl solutions, tetradentate Sr^2+ ^and SrOH^+ ^complexes capture the gradual sorption edge with increasing pH. The tetradentate Sr^2+ ^complex, (>SOH)_2_(>SO-)_2__Sr^2+^, dominates at near neutral pH where sorption is minimal. As pH and percent sorption increase, the tetradentate hydrolyzed SrOH^+ ^complex, (>SOH)_2_(>SO-)_2__ SrOH^+^, accounts for most of the strontium sorbed to the surface. In solutions with lower ionic strength (0.005 M NaCl), the sorption edge shifts to lower pH and a monodenate Sr^2+ ^complex on the diffuse plane, >SOH...Sr^2+^, is needed to fit the data in addition to the tetradentate strontium complexes. Inclusion of other outersphere complexes (such as >SOH_ Sr^2+^, tetradentate SrCl^+^, and tetradentate SrClOH) failed to capture the enhanced strontium uptake at lower ionic strength. The dominance of the *β*-plane Sr^2+ ^complexes at lower ionic strength over the *β*-plane SrOH^+ ^complexes reflects the interplay between the charge in the *β*-plane and the charge of the surface complexes. As the ionic strength increases the *β*-plane SrOH^+ ^complex becomes more dominant. The position of the surface complexes on the *β*- and diffuse planes suggests that strontium retains some or all of its waters of hydration at the amorphous silica surface and is an outer-sphere complex. The designation of sorbed strontium as outer-sphere is supported by the shift in the sorption edge from pH = 7.2 at I = 0.005 M NaCl to pH = 9 at I = 0.1 M NaCl (Figure 4B,F) and by EXAFS data showing only Sr-O bonding and coordination similar to aqueous Sr from pH 8.5 to 9.9 (Figure 1).

The strontium surface complexation model presented here is consistent with Sverjensky's [16] model fit for other alkaline earth sorption data for amorphous silica. Sverjensky [16] described Ca [50] and Mg [47] sorption data over a range of ionic strengths (0.001 to 0.1 N for Ca and 0.005 to 0.05 N for Mg) for a single surface coverage for each cation. The Ca model used the same type of complexes used to describe strontium sorption [>SOH...Ca^2+^, (>SOH)_2_(>SO-)_2__ Ca^2+^, and (>SOH)_2_(>SO-)_2__ CaOH^+^], and the Mg model used two of the three reactions [>SOH... Mg^2+ ^and (>SOH)_2_(>SO-)_2__ Mg^2+^].

### Strontium sorption to goethite

#### Carbonate-free system

The fitted and experimental results are shown in Figure 5(A–C) and Table 8 for the sorption of strontium to goethite in carbonate-free solutions with total Sr ranging from 10^-5 ^to 10^-3 ^M and 0.1 M NaCl. In the absence of dissolved carbonate, strontium sorption to goethite can be fit with monodentate and tetradentate SrOH^+ ^complexes and a tetradentate binuclear Sr^2+ ^complex all on the *β*-plane:

>SOH + Sr^2+ ^+ H_2_O = >SO-_ SrOH^+ ^+ 2H^+^

4 >SOH + Sr^2+ ^+H_2_O = (>SOH)_2_(>SO-)_2__ SrOH^+ ^+ 3H^+^

4 >SOH + 2Sr^2+ ^= (>SOH)_2_(>SO-)_2__ Sr_2_^2+ ^+ 2H^+^

Combination of reactions 4 and 5 fit most of the experimental sorption data for total strontium concentrations of 10^-5 ^to 10^-3 ^M, however they do not capture the enhanced strontium sorption between pH 7 and 8.5 for total strontium concentrations of 10^-3 ^M (nor did the addition of tetradentate Sr^+ ^or tetradentate SrOHCl complexes). We fit the data with a binuclear tetradentate strontium complex because the abundance of the binuclear strontium complex falls off with decreasing total strontium concentrations due to its second-order dependence on Sr^2+^(Equation 6). Unfortunately, there is no spectroscopic data in this pH range to confirm the presence of a polynuclear strontium complex. If a binuclear strontium complex forms at high surface coverage on goethite, it appears to be fairly unique for alkaline earth cation sorption. Polynuclear complexes were not needed to fit Ca and Mg data [45] with similar surface coverage [16]. Classification of strontium as an outer-sphere complex and its placement on the *β*-plane for all surface complexes is also consistent with EXAFS spectra which show only Sr-O bonding and coordination similar to aqueous strontium indicating that sorbed strontium retains waters of hydration at the mineral surface in carbonate free solutions [26].

The strontium model is consistent with surface complexation reactions fitted to other alkaline earth sorption data to goethite, but could be better refined with data collected over a range of ionic strength. In addition to the mono- and tetradentate MOH^+ ^complexes, Sverjensky [16] found that tetradentate MgOHCl or BaOHNO_3 _surface complexes were needed to fit sorption data at high ionic strength of 0.5 N [45,49] and that a tetradentate M^2+ ^complex was needed to fit sorption of Ca at low surface coverage and low ionic strength [45,52].

### Carbonate system

The fitted and experimental results are shown in Figure 5(D–F) for the sorption of strontium to goethite with total Sr ranging from 10^-6 ^to 10^-4 ^M and 0.1 M NaCl in suspensions with dissolved carbonate. The strontium surface complexation model developed here is based on sorption data from experiments with total Sr from 10^-6 ^to 10^-4 ^M to avoid possible precipitation of strontium carbonate from experiments with the total Sr ~ 10^-3 ^M (data not shown). The base model consists of the tetradentate and monodenate SrOH^+ ^complexes (Equations 4 and 5) and carbonate surface complexation reactions and constants from Villalobos and Leckie [41] adjusted in accordance with the site-occupancy standard state [14] (Table 9) to account for carbonate sorption to goethite:

>SOH + CO_3_^2- ^+ H^+ ^= >SO^-0.2^_COO^-0.8 ^+ H_2_O

>SOH + CO_3_^2- ^+ H^+ ^+ Na^+ ^= >SOCOONa + H_2_O

>SOH + CO_3_^2- ^+ 2H^+ ^= >SOCOOH + H_2_O

The binuclear tetradentate strontium surface complex (Equation 6) was not included in the calculations because its overall contribution is minimal at lower surface coverages (total Sr from 10^-6 ^to 10^-4 ^M) and because it led to convergence problems within FITEQL. Fits to the strontium sorption data require the addition of two monodentate strontium carbonate complexes. In the reactions below we maintain the carbonate stoichiometry and charge distribution between the 0- and *β*-planes as modeled by Villalobos and Leckie [41] for the strontium carbonate complexes.

>SOH + CO_3_^2- ^+ Sr^2+ ^= >SO^0.2-^_COOSrOH^0.2+ ^+ H_2_O

>SOH + CO_3_^2- ^+ H^+ ^+ Sr^2+ ^= >SO^0.2-^_COOSr^1.2+ ^+ H_2_O

Strontium carbonate surface complexes dominate the goethite surface from pH 6 to 10, with the non-carbonate SrOH^+ ^complexes becoming important only at higher pH where less carbonate sorbs to the surface.

The strontium sorption model provides some insight into the pH dependence of SrCO_3 _precipitation at the goethite surface observed using EXAFS [26]. Strontium carbonate precipitate has been identified in the presence of goethite at pH 8.5, but not at pH greater than 8.7 in samples with the same surface loading (total Sr = 10^-3 ^M) and for one sample at pH 9.9 with total strontium = 10^-4 ^M. (Figure 3). Sahai et al [26] concluded that the absence of a strontium carbonate precipitate at pH above 8.7 was due to insufficient carbonate on the surface to nucleate the precipitate, based on a decrease in carbonate sorption on the goethite surface from pH 6 to 10 (using constants from VanGeen et al. [39]). Figure 6A is an example of the amount of carbonate predicted to sorb to the goethite surface using our strontium surface complexation model and measured dissolved carbonate. Strontium carbonate complexes comprise at most 3% of the total amount of carbonate on the surface. The amount of carbonate on the surface has a complex dependence on solution pH. Unlike the linear increase of dissolved carbonate concentrations with pH shown in Figure 6B, the amount of carbonate on the surface increases from pH 6 to 8, reaches a maximum from pH 8 to 9, and sharply decreases with increasing pH where the aqueous carbonate complexes dominate. From pH 6 to 9, there is 10 to 4 times more carbonate on the surface than dissolved in solution, illustrating the high affinity of the goethite surface for carbonate in near-neutral pH suspensions, where surface precipitation of strontium carbonate has been observed [26]. The stoichiometry of the carbonate complexes in our model further suggests that the >SO^0.2-^_COOSr^1.2+ ^may be a precursor to surface precipitation, where as the hydrolyzed carbonate complex is not. Thus strontium carbonate precipitation at the goethite surface is inhibited despite having solutions that are supersaturated at higher pH.

### Extension of strontium surface complexation model to strontium sorption to goethite in NaNO_3 _electrolyte with dissolved carbonate

**Figure 7:**
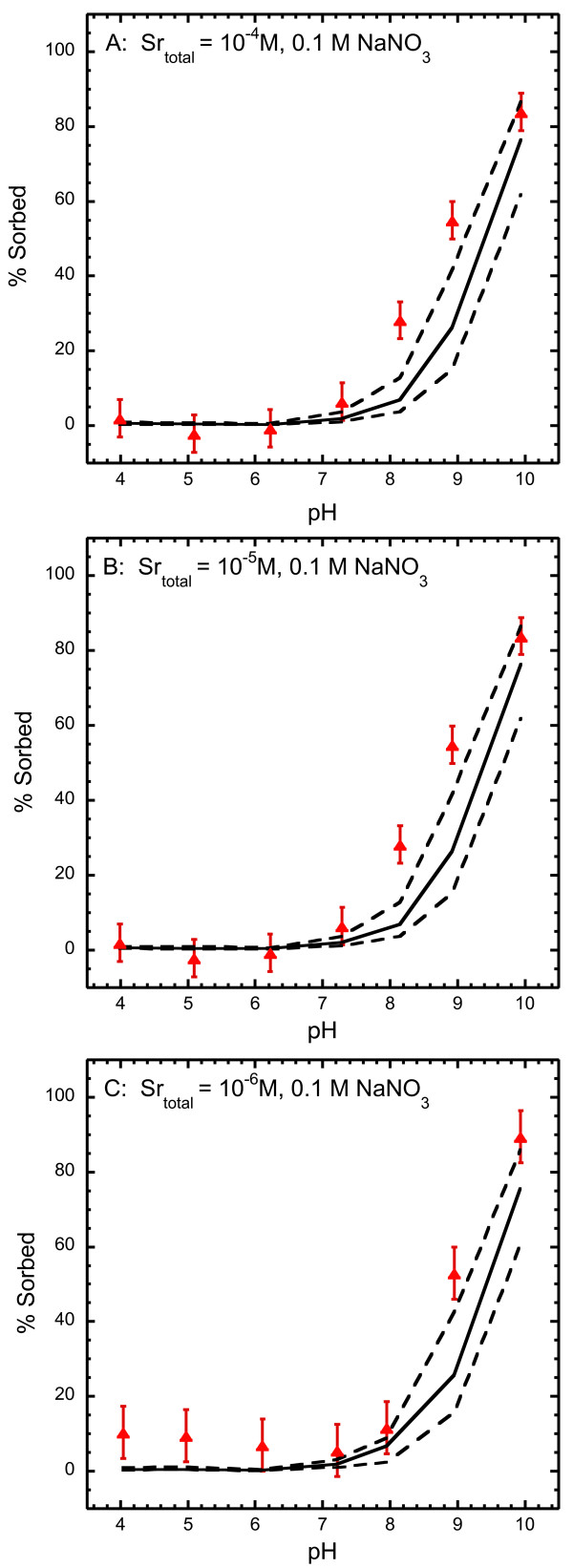
Comparison of experimental and model results for strontium sorption to goethite in 10^-6 ^to 10^-4 ^M Sr(NO_3_)_2 _and 0.1 M NaNO_3 _solutions with dissolved carbonate (A-C). Symbols are the experimental data, solid lines are predictions made with the strontium surface complexation model (Table 9), dashed lines represent ± 0.3 uncertainty in log K for all strontium surface complexation reactions.

Figure 7 compares measured and predicted strontium sorption to goethite in suspensions containing dissolved carbonate, 0.1 M NaNO_3 _and total Sr ranging from 10^-6 ^to 10^-4 ^M. In this figure we show the total amount of strontium sorbed assuming an uncertainty of ± 0.3 log K for the strontium sorption constants (Equations 4, 5, 10, 11). Dissolved carbonate concentrations were estimated from concentrations measured as a function of pH in the NaCl experiments because they were not measured in the NaNO_3 _experiments. The surface complexation model is identical to the strontium carbonate surface complexation model described above with the exception that NO_3_^- ^replaces Cl^- ^for the complexation of the background electrolyte with the surface (Table 9). All constants have been adjusted in accordance with the site-occupancy standard state [14], where the total number of sites equals 16.4 nm^-2^, the solid concentration equals 10 g L^-1 ^and the BET surface area of 27.7 m^2^g^-1^.

Application of the strontium carbonate surface complexation model developed in 0.1 M NaCl goethite suspensions to experiments conducted in 0.1 M NaNO_3 _goethite suspensions is a test of the site-occupancy standard state which allows sorption experiments with varying solid concentrations and specific surface areas to be compared. The solid concentration of the NaNO_3 _experiments was 25% of the solid concentration of the NaCl experiments and the goethite was synthesized from nitrate starting materials resulting in goethite with a surface area that was 75% of the goethite surface area synthesized from chloride starting materials. The strontium carbonate model slightly underpredicts the strontium sorption in NaNO_3 _goethite suspensions assuming an uncertainty of ± 0.3 log K suggested for surface complexation models [16].

### Predictive model

Sverjensky and colleagues have applied Born solvation and crystal-chemistry theory using a site-occupancy standard state to develop a predictive model for surface protonation, alkali, heavy metal, alkaline earth, and anion sorption [6-16]. In this section we augment and re-calibrate Sverjenksy's [16] model for the prediction of alkaline earth speciation sorbed on oxide surfaces by including the fitted strontium surface complexation reactions for goethite and amorphous silica from this study.

Application of Born solvation and crystal-chemistry theory to metal sorption assumes that the standard Gibbs free energy of sorption (ΔG^*θ*^_r,m_) depends on contributions from ion solvation (ΔG^*θ*^_s,m_), electrostatic interactions between the sorbing ion and the surface sites (ΔG^*θ*^_ai,m_), and contributions specific to the sorbing ion (ΔG^*θ*^_ii,m_):

ΔG^*θ*^_r,m _= ΔG^*θ*^_s,m _+ ΔG^*θ*^_ai,m _+ ΔG^*θ*^_ii,m_

such that a given surface complexation equilibrium constant (Log K^*θ*^_r,m_) can be expressed as:

Log K^*θ*^_r,m _= -ΔΩ_r,m_/RT*(1/*ε*_s_) - B_m_(s/r_m_) + log K"_ii,m_

The first term on the right hand side of equation 13 accounts for ion solvation, where ΔΩ_r,m _is the Born solvation coefficient for the r^th ^reaction containing the metal m and *ε*_s _is the dielectric constant for the solid. The second term accounts for the repulsive interaction between the sorbing ion and near surface species, where s is the Pauling's bond strength of the metal-oxygen bonds in the bulk mineral, r_m _is the distance the sorbing ion is repulsed by the underlying metal in the solid due to short-range electrostatic interactions, and B_m _is a constant characteristic of the surface reaction. The final term represents interactions intrinsic to the sorbing ion as well as solvation contributions from the interfacial dielectric constant and the electrostatic attractive interactions. Equation 13 can be reduced to:

Log K^*θ*^_r,m _= -ΔΩ_r,m_/RT*(1/*ε*_s_)+log K''_ii,m_

for a given surface reaction if the repulsive interactions in the electrostatic term are minimal. Linear regressions of log K^*θ*^_r,m _versus 1/*ε*_s _yield a slope equal to -ΔΩ_r,m_/RT and a y-intercept equal to log K"_ii,m _(or B_m_(s/r_m_) + log K"_ii,m _if repulsion interactions are important). This regression serves as a fundamental calibration for the predictive model, because it allows the equilibrium constant for a given surface reaction to be estimated for solids of varying dielectric constants.

In the absence of enough data to calibrate Equation 14, surface equilibrium constants can be calculated with ΔΩ_r,m _and log K"_ii,m _derived from two additional regressions (Equations 15 and 17). Unknown values of ΔΩ_r,m _can be estimated from the absolute solvation coefficient of a given surface complex, Ω_abs r_, and the effective electrostatic radius of the sorbing ion, R_e,m_. The Ω_abs r _is estimated from a regression of known ΔΩ_r,m _versus R_e,m_:

ΔΩ_r,m_= *η*/R_e,m _- Ω_abs r_

where *η *= 166.027 kcal Å mol^-1 ^[8]. R_e,m _is a function of the hydrated radii, r_m,hydr_, [55] and an empirical constant specific to the sorbed species, *γ*_m_:

R_e,m _= r_m,hydr _+ *γ*_m_

In the regression analysis *γ*_m _is a variable used to produce a theoretical slope equal to 1 for ΔΩ_r,m _versus *η*/R_e,m_. Similarly, unknown log K"_ii,m _for a given surface complex can be estimated from a linear regression of known values of log K"_ii,m _(the y-intercept in Equation 14 if repulsive interactions between the near surface species and sorbing ion are not important) versus the ion radius, r_x,m_, because log K"_ii,m _is assumed to be intrinsic to the sorbing ion and independent of differing solid properties. The resulting regression is:

Log K''_ii,m _= slope * r_x,m _+ y-intercept

### Model Calibration

In this section we analyze the new strontium surface reactions for amorphous silica and goethite together with other alkaline earth surface reactions to re-calibrate Sverjensky's [16] predictive model for alkaline earth sorption. Strontium surface reactions on goethite and amorphous silica from this study are re-written as:

2>SOH + 2>SO^- ^+ Sr^2+ ^+ OH^- ^= (>SOH)_2_(>SO^-^)_2__SrOH^+ ^(tet_SrOH^+^)

2>SOH + 2>SO^- ^+ Sr^2+ ^= (>SOH)_2_(>SO^-^)_2__Sr^2+ ^(tet_Sr^2+^)

>SO^- ^+ Sr^2+ ^+ OH^- ^= >SO^-^_SrOH^+^

>SOH + Sr^2+ ^= >SOH...Sr^2+^

and the equilibrium constants used in the fits are converted from the 1 M standard state (Log K^0^) to the site-occupancy standard state (Log K^*θ*^) using the following equations to be consistent with stoichiometry presented by Sverjensky [16]:

Log K^*θ*^_tet_SrOH+ _= Log K^0^_tet_SrOH+ _+ 2pH_zpc _+ ΔpK^o^_n _+ log C_s_^3^(N_s_A_s_)^4^/(N^†^A^†^)^2 ^+ 14

Log K^*θ*^_tet_Sr2+ _= Log K^0^_tet_Sr2+ _+ 2pH_zpc _+ ΔpK^o^_n _+ log C_s_^3^(N_s_A_s_)^4^/(N^†^A^†^)^2^

Log K^*θ*^_>SO-_SrOH+ _= Log K^0^_>SO-_SrOH+ _+ pH_zpc _+ ΔpK^o^_n _+ log (N_s_A_s_)/(N^†^A^†^) + 14

Log K^*θ*^_>SOH...Sr2+ _= Log K^0^_>SOH...Sr2+ _+ log (N_s_A_s_)/(N^†^A^†^)

**Figure 8:**
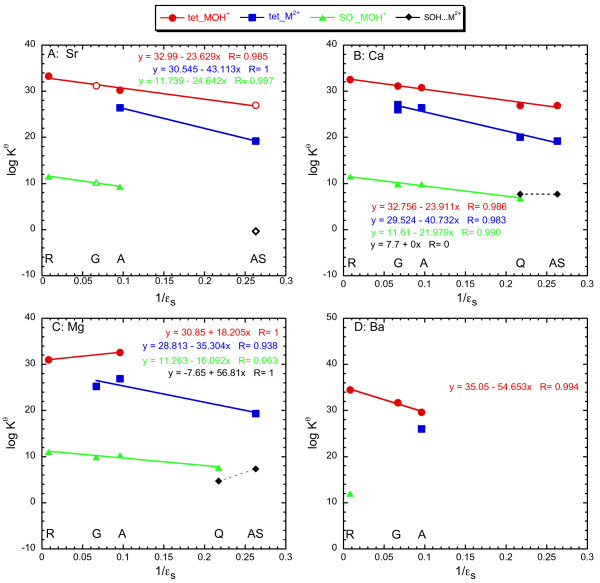
Log K^*θ *^for (>SOH)_2_(>SO^-^)_2__MOH^+^, (>SOH)_2_(>SO^-^)_2__M^2+^, >SO^-^_MOH^+^, and >SOH...M^2+ ^versus the dielectric constants for rutile (R), goethite (G), *γ*-alumina (A), quartz (Q), and amorphous silica (AS). Linear regressions are shown as lines of the same color as the data. Values of log K^*θ*^_r,m _for reactions involving Sr and goethite or Sr and amorphous silica are from this study and are shown as open symbols, all other log K^*θ*^_r,m _are from Sverjensky 2006 and are shown as solid symbols. The ''tet'' prefix refers to tetradentate surface sites.

**Figure 9:**
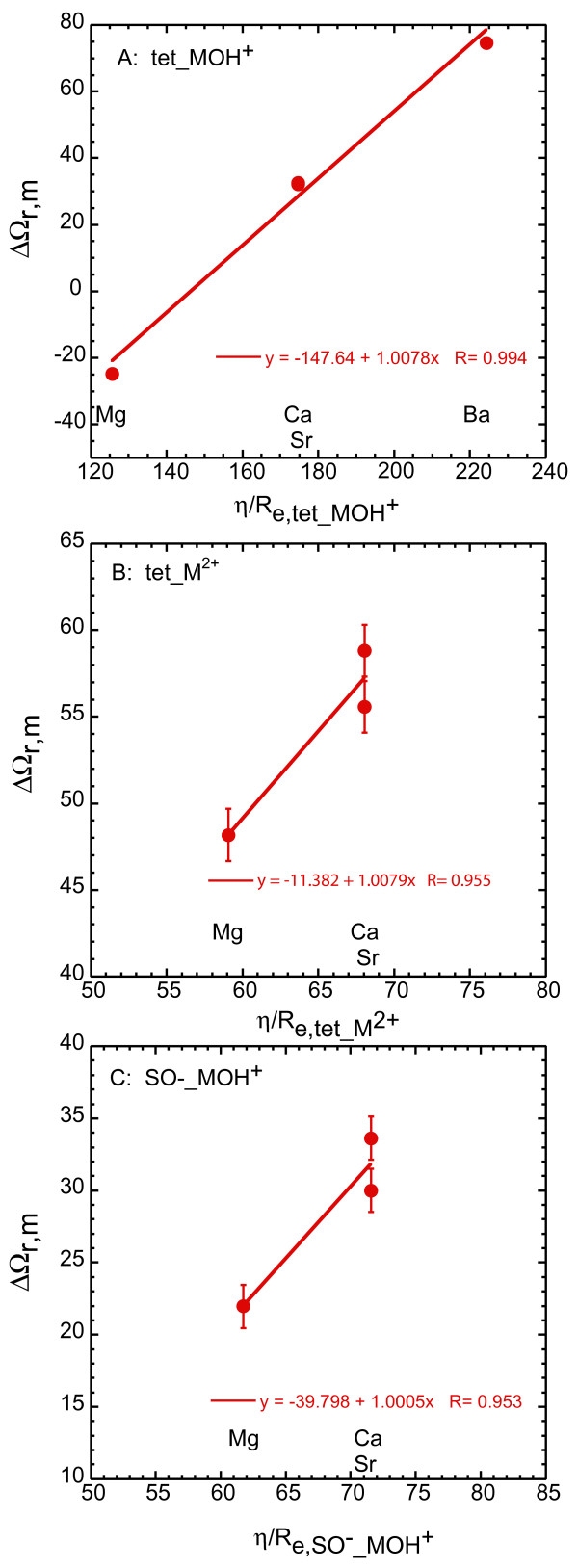
Born solvation coefficient (ΔΩ_r,M_) versus the effective electrostatic radii (*η*/R_e,m_) for Sr, Ba, Ca and Mg for A: (>SOH)_2_(>SO^-^)_2__MOH^+^, B: (>SOH)_2_(>SO^-^)_2__M^2+^, and C: >SO^-^_MOH^+^. Values for ΔΩ_r,M _are derived from regression slopes in Figure 8.

We cannot evaluate the binuclear and strontium carbonate surface reactions with this model, because data are insufficient for calibration (Equations 6–11). Table 10 lists the surface equilibrium constants (Equation 18a-21a) from fits to Sr sorption to goethite and amorphous silica from this study and from fits for other alkaline earth cations [16]. The regression of log K^*θ*^_r,m _versus 1/*ε*_s _for the formation of Sr, Ca, Mg, and Ba surface reactions are linear with correlation coefficients of 0.938 ≤ R ≤ 0.997 with at least three data points (Figure 8, Table 10). We include regressions for Ca, Mg and Ba in Figure 8 because the slopes and y-intercepts used in the calibration of the model are slightly different than those reported in Sverjensky [16] and to provide the reader with snapshot of the data used to calibrate the model. Table 11 lists ΔΩ_r,m _and log K"_ii,m _derived for Sr and for Ca, Mg, and Ba from the regressions in Figure 8 (Equation 14), Ω_abs r _and fitting parameter *γ*_i _from regressions of ΔΩ_r,m _and *η*/R_e,m _in Figure 9 (Equation 15 and 16). Our calculations differ from Sverjensky [16] only in our use of alkaline earth hydration radii from Robinson and Stokes [55] for r_m,hydr_. In some cases Sverjensky [16] used r_m,hydr _as a fitting parameter to achieve reasonable linear regressions. We chose not do this because it introduces a second fitting parameter in addition to *γ*_i_, which is adjusted to yield a theoretical slope equal to one in Equation 15. Additionally, we observe identical values of ΔΩ_r,m _for the formation of (>SOH)_2_(>SO^-^)_2__CaOH^+ ^and (>SOH)_2_(>SO^-^)_2__SrOH^+ ^which is consistent with the two species having the same effective hydration radius. There is a greater difference in ΔΩ_r,m _for the formation of (>SOH)_2_(>SO^-^)_2__M^2+ ^and >SO-_MOH^+ ^for Ca and Sr; however it is only slightly greater than an uncertainty of ± 1.5 ΔΩ_r,m _[16].

**Table 10:** Equilibrium constants (log K^θ^) for alkaline earth surface complexation reactions.

Solids	ε	log K^θ^_Ca_	log K^θ^_Mg_	log K^θ^_Sr_	log K^θ^_Ba_
2>SOH + 2>SO^- ^+ M^2+ ^+ OH^- ^= (>SOH)_2_(>SO^-^)_2__MOH^+^					
rutile	121	32.5	31	33.3	34.5
goethite	15	31.1		^a^31.2	31.7
γ-Al2O3	10.4	30.8	32.6	30.2	29.6
quartz	4.6	26.9			
amorphous silica	3.8	26.9		^a^27.0	

2>SOH + 2>SO^- ^+ M^2+ ^= (>SOH)_2 _(>SO^-^)_2__M^2+^					
goethite	15	27.1			
goethite	15	26	25.2		
γ-Al2O3	10.4	26.4	26.9	26.4	26
quartz	4.6	20			
amorphous silica	3.8	19.2	19.3	^a^19.2	

>SO^- ^+ M^2+ ^+ OH^- ^= >SO^-^_MOH^+^					
rutile	121	11.5	11	11.5	12
goethite	15	9.8	9.9	^a^10.2	
γ-Al2O3	10.4	9.8	10.3	9.3	
quartz	4.6	6.8	7.6		
amorphous silica	3.8				

>SOH + M^2+ ^= >SOH...Sr^2+^					
quartz	4.6	7.7	4.7		
amorphous silica	3.8	7.7	7.3	^a^-0.4	

^a^Values for Sr sorption to amorphous silica and goethite are from this study. All other values are from Sverjensky [16]

**Table 11:** Regression slopes and intercepts from Figure 8 used to calibrate predictive alkaline earth surface complexation model.

**Alkalin Earth**	**slope**	**r_m_**	**log K"_ii,m _(expt)**	**η/R_e,m_**	**ΔΩ_r,m _(expt)**	**r_hydr,m_**	**γ_j_**	**Ω_abs_**
2>SOH + 2>SO^- ^+ M^2+ ^+ OH^- ^= (>SOH)_2_(>SO^-^)_2__M^2+^								
Mg	18.21	0.72	30.85	125.78	-24.84	3.46	-2.14	147.64
Ca	-23.91	1.00	32.76	174.77	32.62	3.09	-2.14	147.64
Sr	-23.63	1.16	32.99	174.77	32.24	3.09	-2.14	147.64
Ba	-54.65	1.36	35.05	224.36	74.57	2.88	-2.14	147.64

2>SOH + 2>SO^- ^+ M^2+ ^= (>SOH)_2_(>SO^-^)_2__M^2+^								
Mg	-35.30	0.72	28.813	59.08	48.17	3.46	-0.65	11.38
Ca	-40.73	1	29.524	68.04	55.57	3.09	-0.65	11.38
Sr	-43.11	1.16	30.545	68.04	58.82	3.09	-0.65	11.38
Ba		1.36		74.45		2.88	-0.65	11.38

>SO^- ^+ M^2+ ^+ OH^- ^= >SO^-^_MOH^+^								
Mg	-16.09	0.72	11.26	61.72	21.96	3.46	-0.77	39.8
Ca	-21.98	1.00	11.61	71.56	29.99	3.09	-0.77	39.8
Sr	-24.64	1.16	11.74	71.56	33.62	3.09	-0.77	39.8
Ba		1.36		78.68		2.88	-0.77	39.8

**Figure 10:**
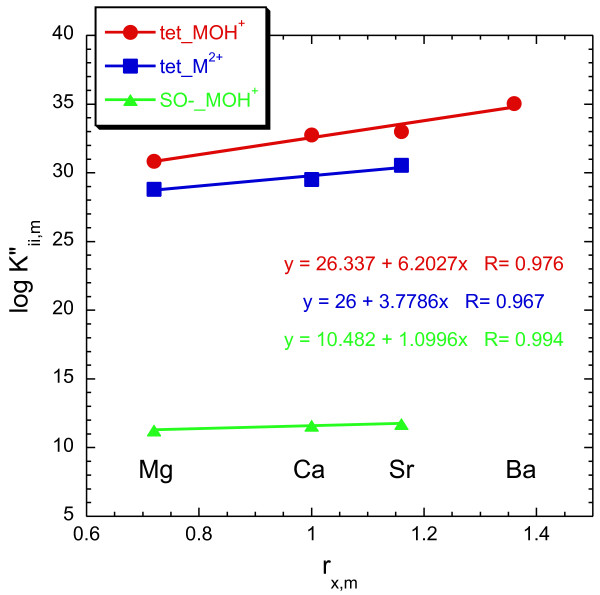
Regression of log K"_ii,m _versus r_x,m _for the formation of (>SOH)_2_(>SO^-^)_2__MOH^+^, (>SOH)_2_(>SO^-^)_2__M^2+^, and >SO^-^_MOH^+^. Log K"_ii,m _are derived from regression y-intercepts in Figure 9. The "tet" prefix refers to the tetranuclear surface sites.

Figure 10 is a plot of the log K"_ii _for (>SOH)_2_(>SO^-^)_2__MOH^+^, (>SOH)_2_(>SO^-^)_2__M^2+ ^and >SO^-^_MOH^+ ^versus ion radii (Equation 17). The linear trends (R > 0.97) support the notion that repulsive interactions between alkaline earths and metals in the underlying substrate are minimal and the y-intercept in Figure 9 represents log K"_ii,m _for all three surface species. Sverjensky [16] estimated the repulsive interactions for M^2+ ^surface reactions, but assumed they were insignificant for surface reactions involving MOH^+^. Results from EXAFS analyses indicate that strontium sorbs mostly as a hydrated surface complex, which is consistent with a large separation between the sorbed cation and metal cations in the substrate and thus minimal repulsion.

Our strontium surface reaction model fits nicely within the constraints of Sverjensky's [16] larger alkaline earth sorption model based on the regression analysis (Figures 8, 9, 10). The good correlation between Log K^*θ*^_r,Sr _versus 1/*ε*_s _for the formation of >SO^-^_SrOH^+ ^and (>SOH)_2_(>SO^-^)_2__SrOH^+ ^surface complexes suggests that equilibrium constants for these reactions can be predicted for many solids with varying dielectric constants. It is also possible to estimate equilibrium constants for (>SOH)_2_(>SO^-^)_2__Sr^2+ ^for other solids even though the regression is based only on two solids, because *γ*-alumina (*ε*_s _= 10.4) and amorphous silica (*ε*_s _= 4.6) span a fairly wide range in dielectric constant. It is not possible to estimate the formation of >SOH...Sr for a wide range of solids, because data are available only for amorphous silica (I = 0.005 N NaCl). Estimates of ΔΩ_r,m _for the formation of the diffuse layer species (>SOH...M^2+^) from ΔΩ_r,m _and a theoretical slope equal to one did not reproduce the limited data for this reaction. This species appears to be a very important complex for the uptake of alkaline earths on amorphous silica (and perhaps quartz) at low ionic strength, but it doesn't appear to be important for other oxides and hydroxides based on the available data.

**Figure 11:**
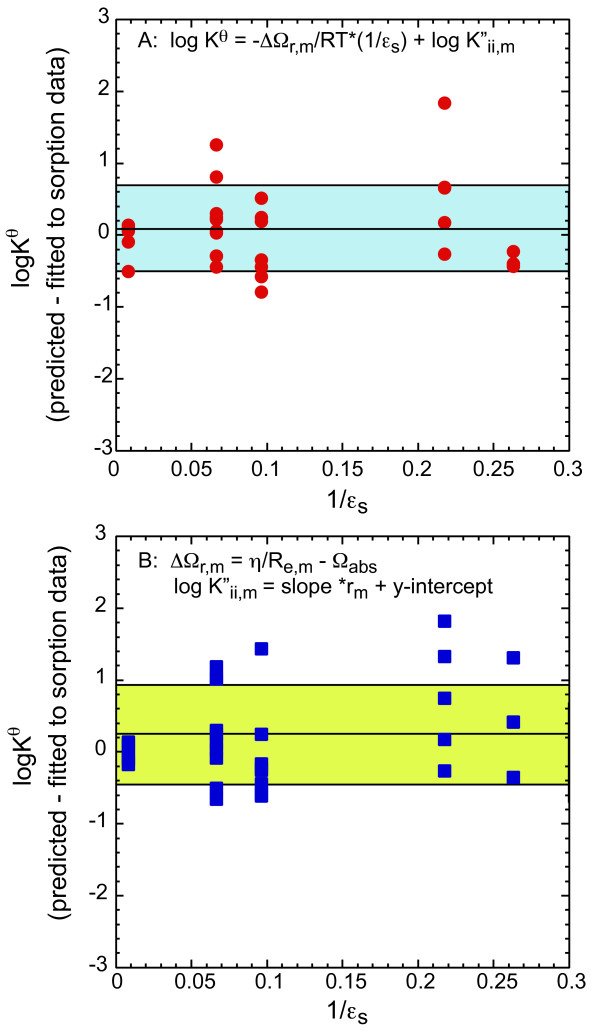
Comparison of log K^*θ*^_r,m _values fitted to sorption data and predicted from Born solvation and crystal chemistry theory using (A) regressions of log K^*θ*^_r,m _= -ΔΩ_r,m_/RT*(1/*ε*_s_) + log K''_ii,m _(Equation 14) and (B) substitution of estimated ΔΩ_r,m _and log K''_ii,m _from Equations 15 and 17 to solve for log K^*θ*^_r,m _= ΔΩ_r,M_/RT*(1/*ε*_s_) + log K''_ii,m _(Equation 14). Colored boxes correspond to standard deviation of 0.6 and 0.7 log K units in A and B respectively.

Figure 11 compares the difference between equilibrium constants for the formation of (>SOH)_2_(>SO^-^)_2__MOH^+^, (>SOH)_2_(>SO^-^)_2__M^2+ ^and >SO^-^_MOH^+ ^fitted to sorption data and predicted directly by the regression of Log K^*θ*^_r,m _vs 1/*ε*_s _(Equation 14) and by substitution of estimated values of ΔΩ_r,m _and log K"_ii,m _into Equation 14 from regressions in Equations 15 and 17. We compare predicted constants from regression of Equation 14 if the surface reaction is calibrated with three or more different solids. On average the regression over predicts Δlog K^*θ*^_r,m _by 0.1 ± 0.6 (1*σ*). Prediction of all fitted equilibrium constants using regressed values for ΔΩ_r,m _and log K"_ii,m _(Equation 15 and 17) substituted into Equation 14 yield an average log K^*θ *^= 0.2 ± 0.7 (1*σ*). The overall uncertainty of the predicted model appears to be about twice that of log K^*θ *^values fitted to sorption data (log K^*θ *^± 0.3).

## Conclusion

Strontium sorption to amorphous silica and goethite can be modeled as a series of outer-sphere SrOH^+ ^and Sr^2+ ^complexes at tetradentate and monodentate surface sites. Reaction stoichiometry for strontium sorption is consistent with that used to model sorption of other alkaline earth metals [16] and allows strontium sorption to be evaluated over a wide range of solids in waters of varying composition. Surface equilibrium constants fit the sorption data to ± 0.3 log K units over a wide range of strontium surface coverage (total Sr = ~10^-6 ^to 10^-3^M) in the presence and absence of dissolved carbonate. There are two key differences between strontium sorption to amorphous silica and goethite. Amorphous silica requires the formation of Sr^2+ ^at the diffuse plane to account for enhanced sorption at low ionic strength, where as goethite does not. Dissolved carbonate does not appear to sorb to amorphous silica or impact the uptake of strontium to its surface, where as significant amounts of carbonate sorb to goethite and suggest the formation of strontium carbonate surface complexes to account for much of strontium uptake to goethite.

The overall alkaline earth model together with its predictive capability suggests that an additive approach can be used to describe sorption reactions in complex geochemical environments. The regression analysis done here suggests that alkaline earth sorption is largely a function of the solvation of the sorbing cation with minimal contributions between the sorbing cation and metals in the substrate as would be expected for outer-sphere sorption. Although the model can predict equilibrium constants for three non-carbonate surface reactions to within ± 0.7 log K, calibration is still fairly limited. There is a need for experimental data over wider range in ionic strength to determine the importance of *β*-plane MOHL and diffuse-plane M^2+ ^surface species. It is also important to determine the role that carbonate and sulfate play on metal sorption to iron hydroxides and other oxides because both anions are abundant in the Earth's surface environment and will play a large role in the mobility of contaminants.
